# Computational approaches for flavivirus drug discovery: targeting structural and non-structural proteins

**DOI:** 10.1039/d6md00108d

**Published:** 2026-05-22

**Authors:** Mayasah Al-Nema, Mariana Manzano Rendeiro, Daniella Liao, Marwan Abdelmahmoud Abdelkarim Maki

**Affiliations:** a School of Biomedical Engineering, University of British Columbia Vancouver BC Canada mayasah.alnema@ubc.ca mayasahalnema@gmail.com; b Faculty of Medicine, University of British Columbia Vancouver BC Canada; c Department of Integrative Oncology, BC Cancer Research Centre Vancouver BC Canada; d Faculty of Pharmaceutical Sciences, UCSI University Kuala Lumpur Malaysia

## Abstract

The *Flaviviridae* family comprises some of the most pathogenic viruses associated with global outbreaks and severe health complications. Mosquito-borne viruses such as Zika, Dengue, West Nile, Japanese Encephalitis, and Yellow Fever account for millions of infections annually, particularly in tropical and subtropical regions. Their ability to infect multiple tissues and cause a wide range of clinical manifestations poses a significant global public health challenge. Despite progress in developing therapeutic strategies and vaccines, effective antiviral treatments remain limited, which emphasises the urgent need to identify novel lead compounds that act through distinct inhibitory mechanisms. Understanding the structural and functional organisation of flavivirus proteins, including the capsid, pre-membrane, envelope, and seven non-structural proteins, can facilitate the identification of promising therapeutic targets. However, frequent mutations in these proteins often confer resistance, underscoring the need for more robust antiviral strategies. Computational approaches offer powerful tools for accelerating antiviral drug discovery by enabling accurate prediction of biological activity and toxicity from chemical structures. Integrating these methods into drug development pipelines enables the rapid identification and optimisation of promising candidates. Accordingly, this review discusses recent applications of computational techniques in the discovery of flavivirus inhibitors, highlighting key examples and emerging trends from recent studies. It also outlines current challenges and future directions in advancing flavivirus drug discovery.

## Abstract 2

Schematic overview of an integrative computational pipeline for drug discovery.

## Graphical abstract

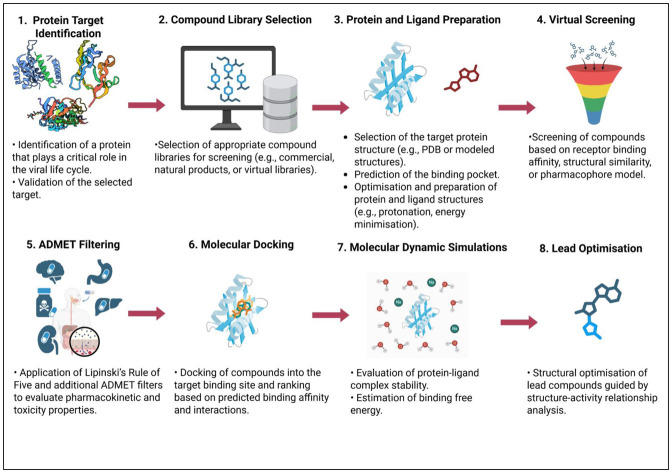

## Introduction

Viral infections are a major cause of morbidity and mortality worldwide, contributing significantly to the growing global healthcare burden.^1^ As obligate intracellular pathogens, viruses can infect a wide range of hosts and are often transmitted from animal reservoirs into human populations.^2^ Novel viruses pose a serious threat to humans when several conditions are met. 1) Direct contact between humans and the animal reservoir; 2) the virus has or develops the ability to transmit from human to human; 3) and such transmission enables the virus to expand its geographical range beyond the initial area of spread. When these conditions are met, viral infections constitute a major risk to human health due to their rapid and widespread transmission, as well as their ability to undergo genetic modification.^3^ Viruses are responsible for several potentially severe illnesses, including hepatitis B and C, AIDS, encephalitis, avian flu, severe acute respiratory syndrome, and coronavirus disease 2019. Among these, flaviviruses are a genus of mosquito-borne viruses that cause life-threatening infections in humans. This class consists of more than 70 types, including the most recognised Zika virus (ZIKV), dengue virus (DENV), West Nile virus (WNV), Japanese encephalitis virus (JEV), and yellow fever virus (YFV).^4^

Flaviviruses are associated with a broad spectrum of diseases that can be categorised into two phenotypes: systemic illnesses, ranging from asymptomatic or mild fever to severe haemorrhagic manifestations, as observed with DENV and YFV, and neurological complications associated with ZIKV, JEV, and WNV. According to the World Health Organisation (WHO), mosquito-borne flaviviruses cause millions of human infections and thousands of deaths annually, and their transmission is further exacerbated by urbanisation, population growth, and climate change.^5^ Despite being a major global health threat, therapeutic options for flavivirus infections remain limited.^6^ Although vaccines against some viruses are available, their effectiveness has been variably successful due to the long-term viral persistence in vector populations and potential of re-emergence.^7^ Several antiviral drugs are currently available, such as acyclovir, which has been studied for its potential to combat both hepatitis B and C. However, despite their widespread application, these drugs exhibit risks, particularly for younger patients. In addition, chemical treatments are commonly used but often lack consistency. For instance, ribavirin, a nucleoside analogue used to treat respiratory syncytial virus (RSV) and hepatitis C, has inconsistent outcomes due to toxicity and limited efficacy.^8^ Therefore, there is an urgent need for the development of novel, effective, and targeted antiviral therapies.

In recent decades, computational chemistry techniques, such as computer-aided drug design (CADD), have emerged as innovative approaches for discovering and designing therapies for a wide range of diseases.^7,9^ These approaches accelerate the identification of novel compounds, thereby reducing synthesis costs and saving valuable time in drug development.^10^ The structure-based drug design technique utilises the 3D structure of a biological target to develop new drug molecules, while the ligand-based drug design method correlates the chemical structure of a molecule with its physical, pharmaceutical, biochemical, and biological effects, thereby enabling the prediction of molecular activity and toxicity.^11^ Designing antiviral compounds that selectively target specific receptors is expected to significantly improve drug efficacy by enhancing selectivity towards infected cells while reducing off-target effects, thereby minimising toxicity and maximising therapeutic benefit. However, certain challenges are associated with effectively treating and eliminating viral infection from the host. For example, protected sites such as the blood–brain and blood–testis barriers can act as viral reservoirs, allowing replication at reduced levels and limiting the effectiveness of conventional therapies. Nevertheless, the CADD approach holds promise for identifying compounds that can penetrate these barriers, offering a potential solution to this challenge. In this review, the application of computational methods to identify and design antiviral compounds, with a focus on the *Flaviviridae* family, will be discussed. In addition, recent advances in the use of CADD techniques to treat ZIKV, DENV, WNV, JEV, and YFV will be highlighted, supported by relevant examples.

## Structure and function of flavivirus proteins in viral infection

**Fig. 1:**
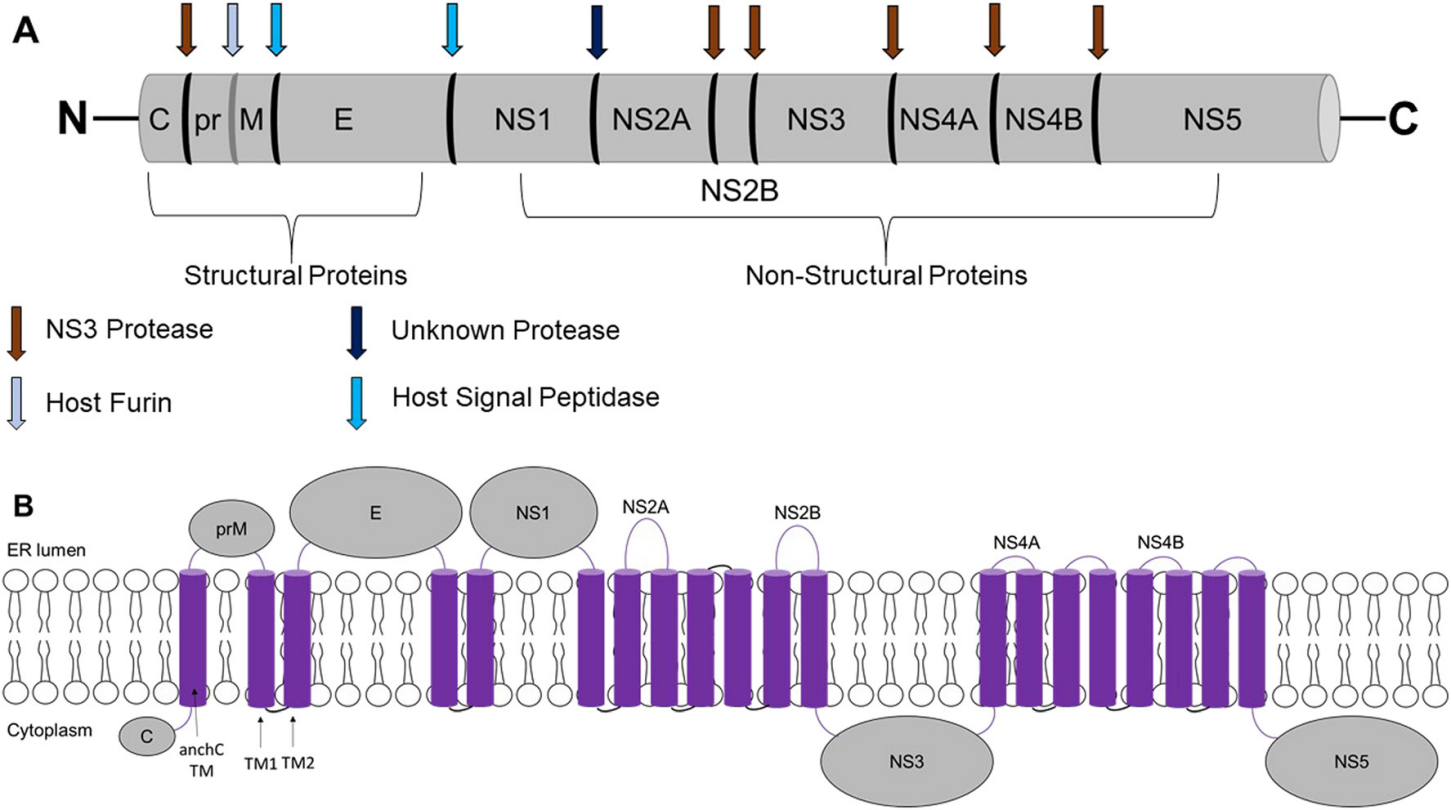
Schematic representation of the flavivirus genome and polyprotein. A) The flavivirus genome is translated into a single polypeptide, which is cleaved by proteases into three structural proteins and seven non-structural (NS) proteins. The structural proteins include capsid (C), premembrane (prM), and envelope (E) proteins, while the NS proteins include NS1, NS2A, NS2B, NS3, NS4A, NS4B, and NS5. Cleavage sites indicated by brown arrows are cleaved by viral proteases, and the sites indicated by blue arrows are cleaved by the host. B) Topology of the flaviviral polyprotein and its transmembrane (TM) domains. The polyprotein is integrated into the endoplasmic reticulum (ER) membrane, with C, NS3, and NS5 located on the cytoplasmic side; prM, E, and NS1 on the luminal side; and NS2A, NS2B, NS4A, and NS4B have multiple TM domains spanning across the ER.

Flaviviruses are enveloped, positive-sense, single-stranded RNA viruses with a diameter of 50 nm and a genome length ranging from 9.4 to 13 kb that encode one open reading frame flanked by a 5′-terminal and lack the poly(A) tail of the 3′-terminal end.^12^ The RNA is translated into a single polypeptide that is cleaved by viral and host proteases into three structural proteins: capsid (C), premembrane (prM), and envelope (E) proteins; and seven non-structural (NS) proteins: NS1, NS2A, NS2B, NS3, NS4A, NS4B, and NS5 proteins (Fig. 1). The structural protein, C protein, which contains ∼120 amino acids, is associated with the virus assembly by packaging the RNA genome and the fusion process. The E protein, which includes ∼495 amino acids, mediates the attachment of viral particles to the host cell receptors. While prM, consisting of ∼165 amino acids, is important in the last phase of the replication cycle of the virus, where cleavage of the prM protein mediates virus maturation and the production of infective particles.^13,14^

**Fig. 2:**
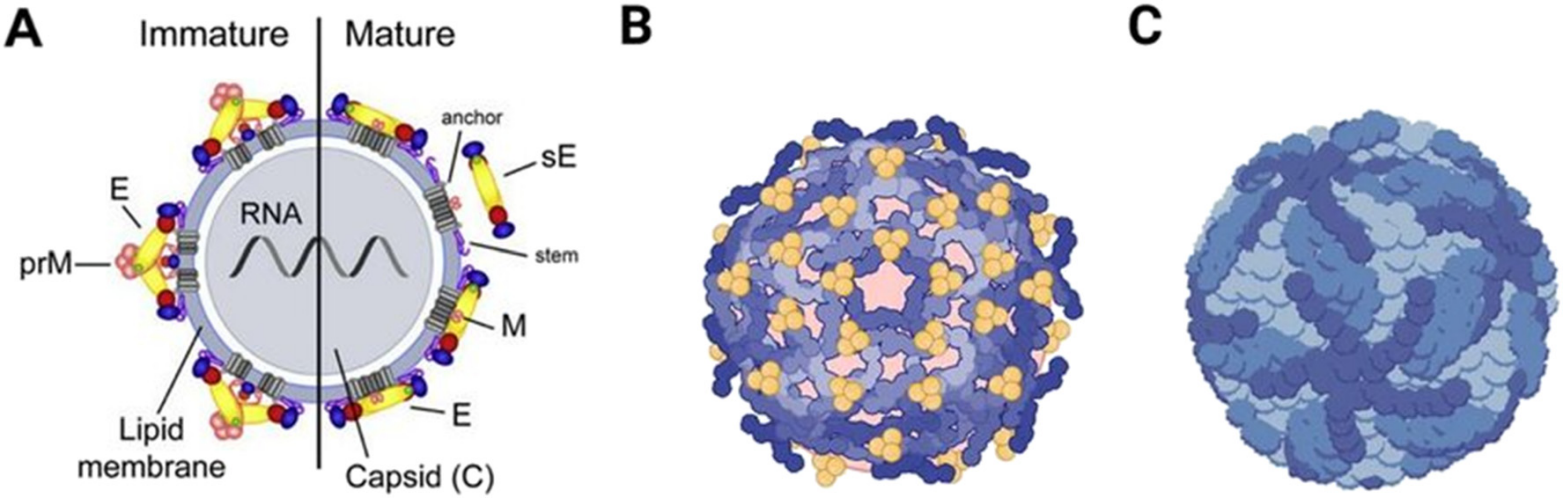
A) Representation of the spiky immature and smooth mature states of flaviviruses. B) Immature virions contain the premembrane protein (prM). C) The mature virions contain membrane (M) and envelope (E) proteins. Reproduced from Gomes da Silva P. *et al. Antibodies*, 2023; under the terms of the Creative Commons CC BY license.^24^

The NS proteins are essential in the replication cycle of flavivirus, evasion of the immune response, and pathogenesis; thus, they are relevant targets for designing antiviral compounds.^15^ NS1 contributes to viral replication and pathogenesis through interactions with host immune proteins.^16^ NS2A is a transmembrane (TM) protein involved in the replication complex formation and suppression of interferon induction, while NS2B functions as a cofactor for the NS3 protease.^17^ NS3 is a multi-domain protein with putative protease activity at its N-terminal serine protease domain. The complexation of NS3 with NS2B is required for the catalytic activity, cleavage of the polyprotein, and unwinding of the secondary structure of the RNA; hence, allowing the synthesis of multiple copies of viral RNA.^18^ In the active conformation of flavivirus, NS2B is wrapped around NS3 and forms part of the enzyme's active site.^19^ While in the inactive conformation, NS2B is partially associated with NS3. Since NS3 is essential to the life cycle of flavivirus, it is considered an attractive drug target for developing antiviral drugs.^20^ On the other hand, the two proteins NS4A and NS4B, together with NS2A and NS2B, act as scaffolds for the replication complex that is essential to viral replication. Whereas the NS5 protein, known as RNA-dependent RNA polymerase (RdRp), is essential for the replication of the RNA.^21,22^ The endoplasmic reticulum (ER) of the host cell is the primary site for the synthesis of structural proteins and assembly of immature, non-infectious flavivirus particles. The arrangement of structural proteins determines the maturation morphologies of flavivirus particles, *i.e.*, spiky (immature, non-infectious) and smooth (mature, infectious) (Fig. 2). Mature flavivirus particles are generated from immature particles in the Golgi lumen *via* two events: proteolytic activity, where prM is cleaved into pr and M by the host furin, and pH-induced conformational change from the radial orientation of pr-E heterodimers to tangential E dimers. As a result, the flavivirus particles are released outside the cell.^23^

## Targeting structural proteins of flaviviruses

Identifying small molecules that selectively inhibit crucial steps in the viral life cycle requires a comprehensive characterisation of the biochemical and structural properties of viral proteins. The structural proteins C, prM, and E undergo a series of conformational changes during the processes of viral entry, assembly, and release.^25^ Immature flavivirus particles become competent for fusion into the host cell membrane after maturation; hence, targeting these structural proteins could prevent infection at an early stage and serve as an effective antiviral strategy. Furthermore, the presence of prM and E proteins on the viral surface enables direct interactions with antibodies and small-molecule inhibitors. Targeting the E protein can block receptor binding, conformational changes, and maturation; inhibiting prM cleavage can prevent virion maturation, and disrupting C protein function can impair viral assembly.^25^

## Capsid (C) protein

**Fig. 3:**
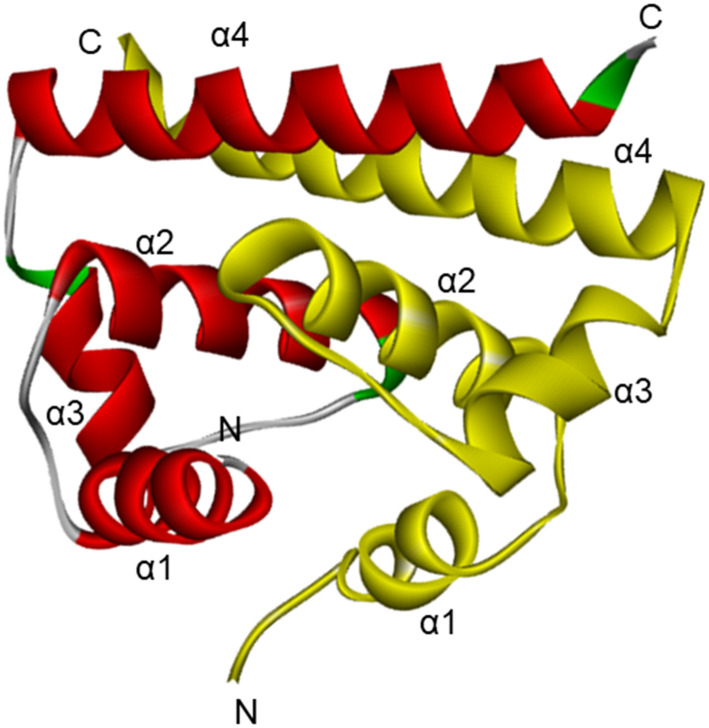
Ribbon diagram of C protein dimer from West Nile virus (WNV). PDB ID: 1SFK.

The highly conserved and basic flavivirus C protein (∼11 kDa) plays multiple roles in the viral life cycle, including viral RNA binding, nucleocapsid formation, and interactions with host cellular proteins that influence metabolism, apoptosis and immune responses.^26,27^ Despite the crucial functions of the C protein, no inhibitor has been identified to date, mainly due to the lack of an active site. Therefore, antiviral strategies should focus on disrupting the capsid interactions with the RNA, host proteins, or C protein–phospholipid membrane. Structurally, the C protein contains an internal hydrophobic region that mediates its association with cellular membranes. It folds into a homodimer, with each monomer comprising four α-helices connected by short loops (Fig. 3). The dimer exhibits an asymmetric charge distribution, where one interface (α2–α2′) mediates lipid droplet interactions and the other interface (α4–α4′), enriched in positively charged residues, binds viral RNA. Capsid dimerisation is promoted by nucleic acid binding and is essential for nucleocapsid assembly.^28^ This structural arrangement provides both flexibility and stability, allowing transient conformations that may regulate interactions with host lipids through autoinhibitory mechanisms. The development of an *in vitro* assembly system for the C protein would be highly beneficial for identifying compounds that disrupt capsid dimerisation or RNA binding, which are key processes to target for antiviral drug development.

## Premembrane/membrane (prM/M)

The prM protein is a key structural component that ensures proper viral maturation by protecting the E protein during virus assembly. In immature virions, prM shields the E protein fusion loop, ensuring proper folding in the acidic *trans*-Golgi network, since premature conformational changes could compromise infectivity.^13^ During maturation, host cell protease furin cleaves the prM domain, yielding two distinct products: the soluble pr peptide, which dissociates upon viral release, and the M protein, which remains embedded in the mature virion's membrane. By inhibiting this cleavage, small molecules can prevent the virus from becoming fully infectious. While no specific inhibitors have been identified to date that directly target prM or block this maturation step, it remains a promising antiviral target for future therapeutic development.^29^

## Envelope (E) protein

The E protein plays an important role in virion assembly, host cell binding, and immune evasion.^12,30,31^ It forms raft-like arrangements of 90 antiparallel homodimers, where each monomer is divided into three domains (EDI–EDIII) connected by flexible hinges that facilitate conformational changes throughout the viral life cycle.^32,33^ E protein contains one or two glycosylated asparagine residues and four conserved histidine residues that mediate cell attachment and regulate the pH-dependent trimerisation and membrane fusion, respectively.^34,35^ The EDI domain, located at the N-terminus, acts as a hinge between EDII and EDIII.^36–39^ It regulates conformational changes in the E protein and stabilises its overall orientation.^40^ It also contains a conserved N-linked glycosylation site at the Asn154 residue, which is important for viral functions.^41^ Therefore, substitutions at this site impair glycosylation, leading to reduced cellular attachment and neurovirulence.^42,43^ A study by Goo *et al.* demonstrated that a single residue substitution in the EDI-EDII hinge region can alter virion stability and neutralisation sensitivity in both WNV and DENV.^34,44,45^

**Fig. 4:**
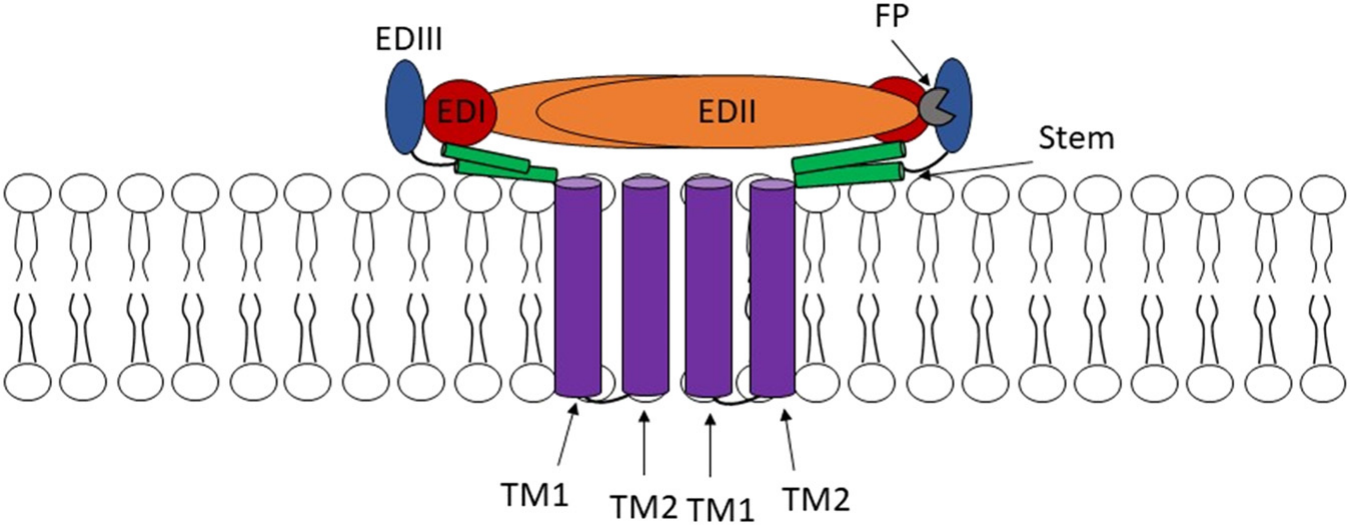
Structure of E protein. Each monomer contains three distinct domains: EDI, red; EDII, orange; and EDIII, blue; a stem region, green; a fusion peptide (FP), grey; and two transmembrane (TM1 and TM2) domains, purple.

The EDII contains a conserved hydrophobic fusion loop (FL), which functions as the internal fusion peptide (FP) embedded in a hydrophobic pocket formed between EDIII and EDI and plays a pivotal role in viral binding and membrane fusion.^46,47^ EDII also mediates the anti-parallel homodimerisation of E proteins. Mutations in this domain disrupt dimer formation, impair viral replication, and attenuate virulence, which underscores EDII's central role in the structural and functional integrity of the virus.^12^ The EDIII is an immunoglobulin-like domain located at the C-terminus and linked to EDI *via* a flexible hinge (Fig. 4). It mediates receptor binding through interactions with host cell surface molecules, such as heparan sulfates, ribosomal protein SA, and low-density lipoprotein receptor-related protein 1 (LRP1).^46^ EDIII also houses key linear and conformational epitopes, many of which are highly conserved across flaviviruses, contributing to both specific neutralisation and cross-reactivity. Mutations within EDIII can alter host cell tropism, virulence, and immune evasion, while some conserved motifs (*e.g.*, JEV's 394HHWH397) serve as minimal epitopes recognised by monoclonal antibodies.^12^ These structural features make EDIII a prime candidate for diagnostic antigen development, vaccine design, and therapeutic targeting, despite antigenic variability and overlapping epitopes with other domains such as EDII.^48^

## Targeting non-structural (NS) proteins of flaviviruses

Although NS proteins are primarily expressed within infected host cells, they are generally not enclosed in the newly formed viruses released from the infected cell, with the exception of NS1, which has been reported to be enclosed within fully assembled flavivirus particles. Compared to the E protein, the highly conserved regions of NS proteins across various flaviviruses act as promising therapeutic targets due to their critical functional roles and selectivity. In general, antiviral strategies have focused on targeting viral replication enzymes, such as protease (NS2B/3), helicase (NS3), and polymerase (NS5). Inhibition or disruption of these proteins interferes with viral replication, making them attractive targets for antiviral drug development.^49^

## NS1

NS1 is considered one of the most promising targets for the development of novel antiviral compounds. It is a highly conserved, multifunctional glycoprotein that plays an essential role in immune evasion and viral replication. It is the key virulence factor that enables the virus to counteract the host's innate immune response, thereby promoting replication within a hostile antiviral environment. NS1 (∼48 kDa) consists of an N-terminal homodimeric RNA-binding domain (RBD), a central effector domain (ED), a short, flexible linker region, and a C-terminal tail, facilitating interactions with viral RNA and host proteins.^50^ Although the ED is dispensable for NS1's biological activity, the RBD is critical, particularly for functions requiring interaction with both viral and host RNAs. This essential role is illustrated by the complete loss of pathogenicity observed when Arg38 and Lys41, two key residues mediating RNA binding, are substituted with alanine.^51,52^ Accordingly, NS1 represents a highly promising antiviral target due to its multifunctional nature and central role in viral pathogenesis and host interaction.

## NS2A

NS2A is a 22 kDa hydrophobic protein essential for replication complex assembly, virion formation, and pathogenesis through interactions with viral RNA, structural proteins, and the NS2B–NS3 protease complex.^53^ NS2A has five integral TM helices and contributes to RNA synthesis through binding to the 3′ untranslated region (UTR). Consequently, mutations in NS2A can disrupt both RNA synthesis and virion assembly.^54^ NS2A also contributes to virion formation by participating in the biogenesis of virus-induced membrane structures. In addition, it suppresses the host's interferon-α/β response, thereby affecting viral replication and inducing apoptosis in infected cells. The NS2A role in evading innate immunity responses further highlights its involvement in disease pathogenesis. A conserved slippery heptanucleotide motif located at the beginning of the NS2A gene mediates a programmed-1 ribosomal frameshift, leading to the production of an extended NS1 variant, NS1′. Mutation of the alanine codon (GCC) at position 30 of NS2A to proline (CCA) disrupts the frameshift-stimulating pseudoknot structure, thereby abolishing NS1′ synthesis.^50^ This functional interdependence demonstrates the essential role of NS2A in the viral replication cycle and highlights its potential as a promising therapeutic target.

## NS3/2B

NS3 protein is a large, ∼70 kDa, multifunctional enzyme with an N-terminal protease domain and a C-terminal helicase domain. It plays a crucial role in viral assembly and pathogenesis. The protease domain contains a catalytic triad comprising His51, Asp75, and Ser135 residues located in a cleft between the β-barrels, as identified in the NS3 protein of DENV4. In contrast, the catalytic activities associated with hydrolysis, RNA 5′-triphosphatase (RTPase), and nucleoside triphosphatase (NTPase) in the helicase domain are mediated by conserved motifs distributed across its three subdomains. The NS3 serine protease activity requires NS2B, a small ∼14 kDa protein, which acts as a cofactor for proper folding and enzymatic activity, forming the functional NS2B–NS3 protease complex. This viral protease mediates cleavages at multiple sites within the viral polyprotein and is indispensable for viral maturation. Mutations or deletions that disrupt NS2B can significantly impact the structural stability of the protease, resulting in autoproteolytic cleavage, *trans*-cleavage, and poor solubility, particularly during crystallisation. Structural studies of flavivirus further reveal that NS2B not only acts as a cofactor but also contributes a β-strand to the NS3 protease domain, which is essential for forming a chymotrypsin-like fold. The C-terminus of NS2B can either be dissociated from NS3 or folded back into a β-hairpin that participates in catalysis.^55,56^ Numerous inhibitors targeting the NS2B–N3 protease complex have been developed for various flaviviruses, showing their potential as a key antiviral target.

## NS4

NS4A and NS4B are membrane-associated proteins that coordinate replication complex formation and drive viral-induced membrane remodelling. NS4A is a small, 16 kDa, TM protein that interacts with NS1, NS3, NS4B, and viral RNA to facilitate RNA release from NS3 helicase and promote formation of replication organelles.^57^ It consists of an N-terminal cytosolic domain and four predicted transmembrane segments (pTMSs). Among these, pTMS1 and pTMS3 fully span the ER membrane, while pTMS2 is embedded within the membrane but does not cross the bilayer. The C-terminal segment, pTMS4, also known as the 2K fragment, spans the ER membrane and acts as a signal peptide that directs NS4B localisation to the ER. The 2K fragment is cleaved from the NS4A-2K-NS4B polypeptide during replication by the NS2B–NS3 protease, releasing mature NS4A.^58^ The NS4A plays a pivotal role in flavivirus-induced membrane remodelling. Heterologous expression of WNV NS4A containing the 2K fragment induces cytoplasmic membrane rearrangements similar to those observed during WNV infection. In contrast, deletion of the 2K fragment impairs NS4A's ability to remodel the membrane and causes its redistribution to the Golgi apparatus. Interestingly, the requirement for the 2K fragment differs in DENV, where proteolytic cleavage of the 2K fragment is necessary for heterologously expressed NS4A to remodel the ER membrane in a manner consistent with DENV infection. These observations indicate that the 2K fragment regulates NS4A-mediated membrane remodelling through virus-specific mechanisms.^59^

On the other hand, the NS4B (∼27 kDa) is a hydrophobic membrane protein, generated by sequential viral and host protease processing and is essential for replication complex stability and membrane organisation. In DENV, NS4B is highly conserved across all four serotypes, sharing more than 85% sequence identity; however, its sequence homology with NS4B from other flaviviruses is lower (∼54%), posing challenges for developing broad-spectrum antiviral inhibitors targeting NS4B.^60^ Several inhibitors targeting NS4B have been developed, including JNJ-A07 and NITD-688, which effectively inhibit DENV replication, highlighting NS4B as an attractive target for antiviral treatment.

## NS5

**Fig. 5:**
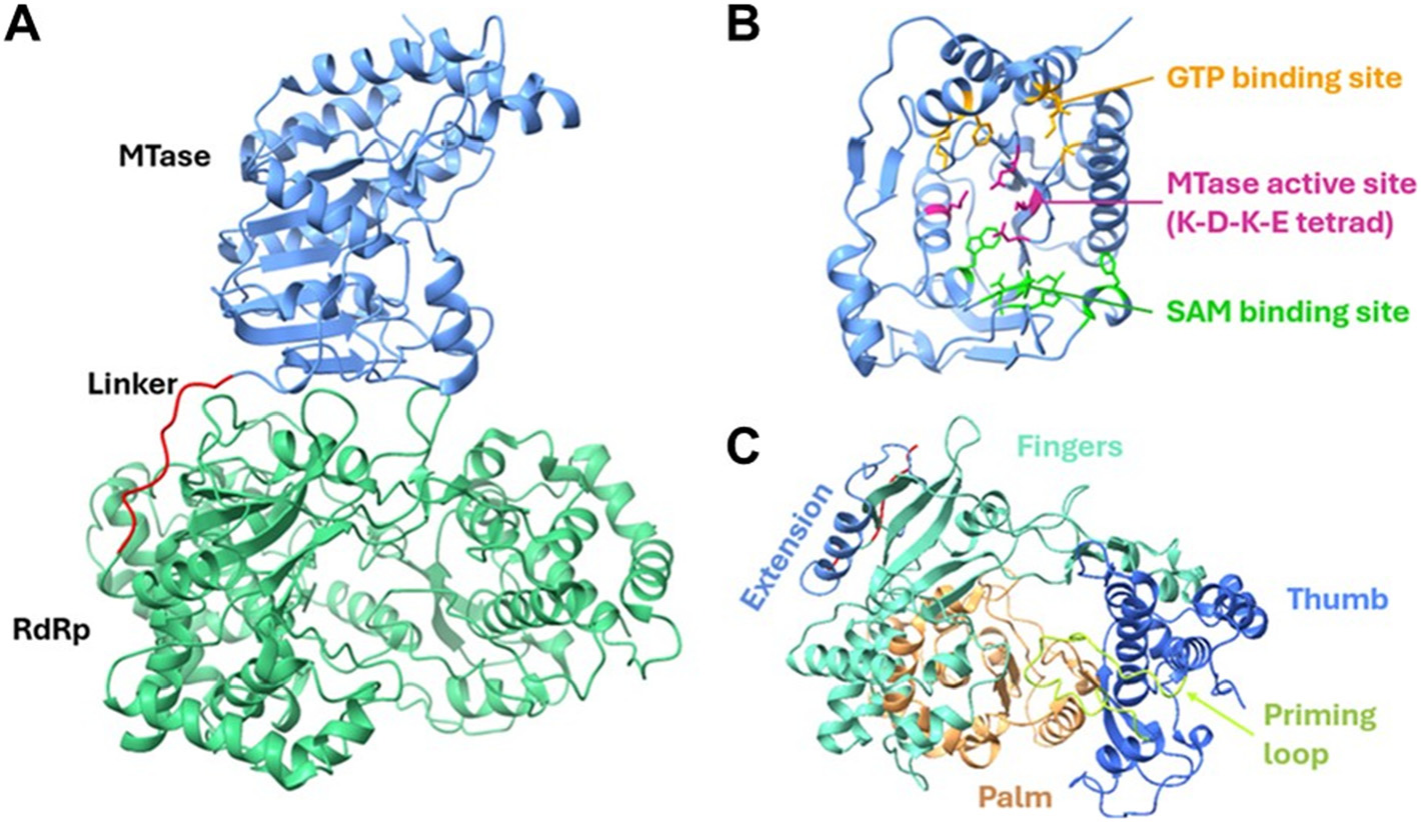
ZIKV NS5 structure (PDB: 5U0B). A) The methyltransferase (MTase) and RNA-dependent RNA polymerase (RdRp) domains are shown in a ribbon representation, illustrating their overall fold. B) Critical residues required for binding guanosine triphosphate (GTP) and *S*-adenosylmethionine (SAM), and MTase catalytic function. C) The RdRp domain is detailed, showing the fingers, palm, and thumb subdomains, the red-coloured linker connecting MTase and RdRp, and the conserved priming loop required for initiating RNA polymerisation. Reproduced from Goh J. Z. *et al.* Vaccines 2024; under the terms of the Creative Commons CC BY license.^61^

NS5 is the largest (∼100 kDa) and most conserved flaviviral protein. It serves as a main enzyme of the viral RNA replication complex. NS5 consists of an N-terminal RNA methyltransferase (MTase) domain and a C-terminal RdRp domain, connected by a flexible linker (Fig. 5A). Mutations in the linker region that impose rigidity reduce the RdRp activity and attenuate the viral replication. The MTase domain can be further subdivided into three distinct subdomains: a helix-turn-helix motif, a β-strand, and an α-helix.^61^ The MTase domain mediates RNA capping by adopting a canonical Rossmann fold architecture, forming the binding sites for both *S*-adenosylmethionine (SAM) and guanosine triphosphate (GTP) (Fig. 5B). At the centre of the MTase domain lies the highly conserved catalytic tetrad K–D–K–E, which is essential for both N-7 and 2′O methylation activities.^62^ The C-terminal subdomain interacts with both the N-terminal and core subdomains to stabilise the MTase structure.^63^ Similar to other viral RdRps, the flaviviral NS5 adopts a characteristic capped “right-hand” architecture comprising three subdomains: the palm, fingers, and thumb (Fig. 5C). The active site is located above the palm domain and is surrounded by structural loops from both the palm and thumb domains, which help stabilise the RNA during polymerisation. To date, all studied flaviviral RdRps initiate RNA synthesis *de novo*.^64^ Consistent with this mechanism, a priming loop within the thumb domain, which contains two conserved aromatic residues, W795 and H798 in DENV RdRp, was found to be critical for *de novo* initiation and is conserved across all flaviviral RdRps.^50^

## *In silico* strategies to identify antiviral inhibitors

**Fig. 6:**
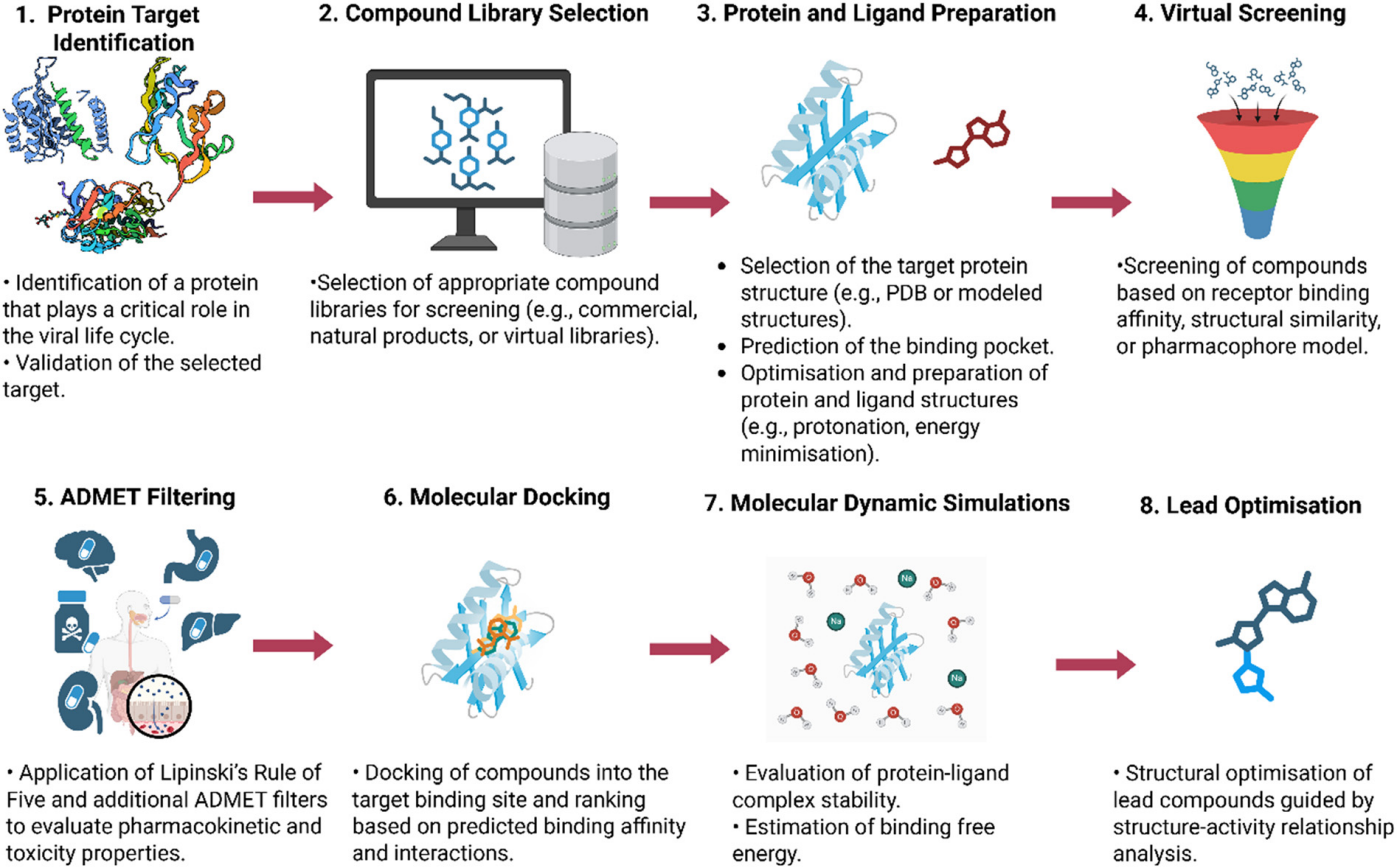
Schematic overview of an integrative computational pipeline for drug discovery.

The detailed structural and functional characterisation of flaviviral proteins provides a critical foundation for rational antiviral drug design. These structural insights enable the identification of key catalytic residues, binding pockets, and conformational features that can be exploited for targeted inhibitor development. Traditionally, the discovery of antiviral drugs relied on *in vitro* screening of large compound libraries. However, these experimental assays are often costly and time-consuming. Consequently, computational approaches have gained prominence as efficient tools to prioritise and filter compounds before experimental validation. These methods can help predict molecular efficacy, thereby facilitating the drug discovery process. Since the emergence of CADD, numerous successful applications have been reported across a wide range of therapeutic targets (Fig. 6).

## Zika virus (ZIKV)

Zika virus (ZIKV) was first discovered and isolated in 1947 from a sentinel rhesus monkey. Initially, it did not attract much attention, as the majority of infections were asymptomatic, while approximately 20% of the infections developed a self-limiting illness with mild symptoms such as fever, headache, rash, and muscle pain.^65^ However, the situation changed in 2014 and 2015, when the virus spread rapidly across the Americas. Emerging evidence revealed that ZIKV infection during pregnancy could cross the placental barrier, infect the foetus, and cause congenital microcephaly by targeting cortical progenitor cells, inducing cell death, and impairing neurodevelopment.^3^ The increased risk of microcephaly associated with the viral infection has resulted in the announcement of ZIKV as a global health emergency by the WHO in 2016. To date, no antiviral drug or vaccine has been approved for the treatment or prevention of ZIKV infection, although extensive efforts are ongoing to develop effective inhibitors.^66^

## Inhibitors of ZIKV

**Fig. 7:**
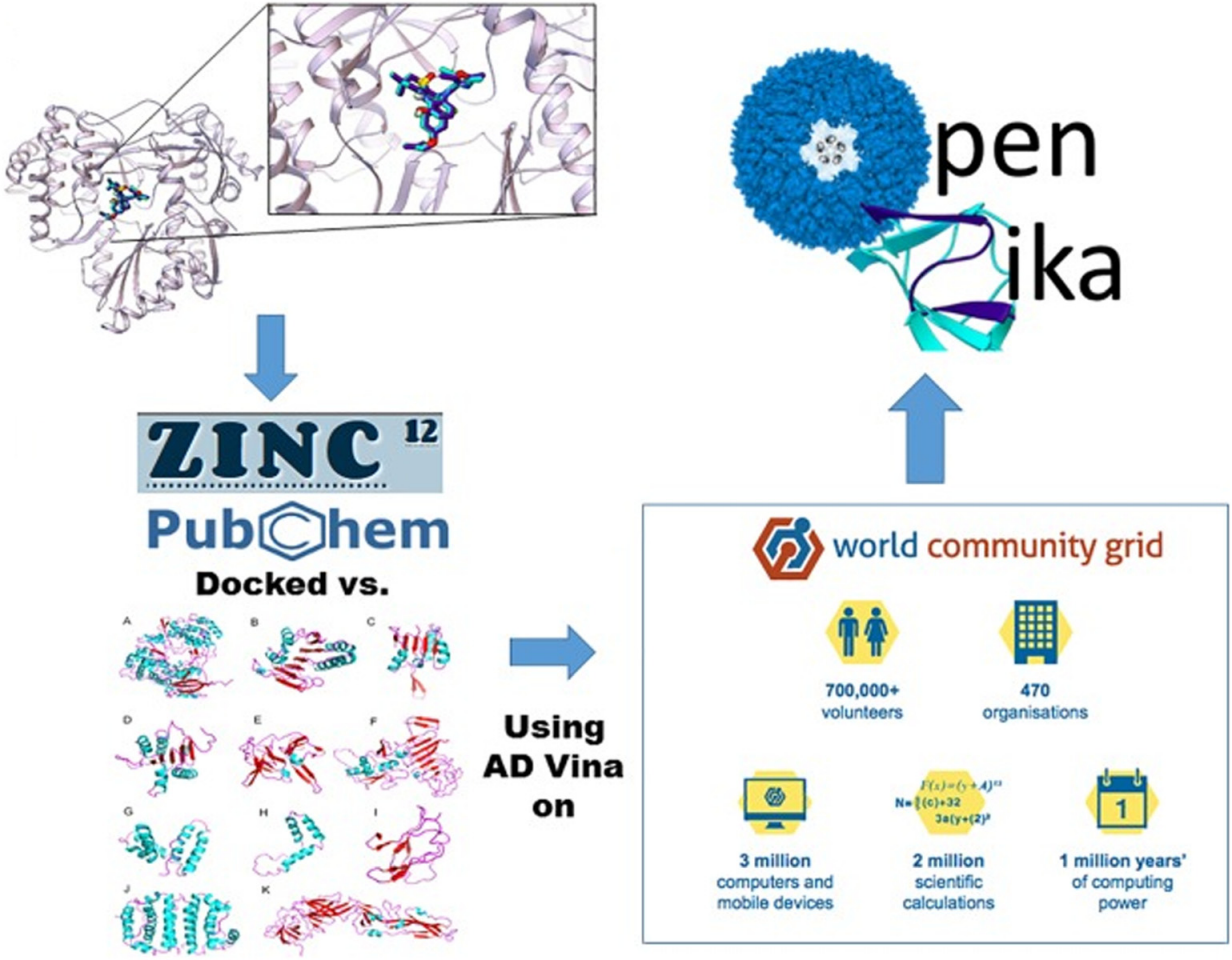
Workflow of the OpenZika project. Millions of compounds from the Zinc database were virtually screened against Zika virus (ZIKV) crystal structures and homology models using molecular docking and QSAR approaches. The top-ranked hits identified through these computational methods were subsequently selected for experimental validation. Reproduced from Ekins S. *et al.* PLOS Neglected Tropical Diseases, 2016; under the terms of the Creative Commons CC BY license.^67^

One of the most successful applications of CADD for discovering ZIKV drug candidates was established by scientists from the United States and Brazil in collaboration with the IBM World Community Grid, known as the OpenZika project. This open science initiative screens tens of millions of drug-like compounds against all available ZIKV homology models and crystal structures using molecular docking and quantitative structure–activity relationship (QSAR) modelling to identify novel hits with potential for anti-ZIKV drug development (Fig. 7). By incorporating multiple protein conformations, this large-scale framework effectively implements an ensemble docking strategy, which is increasingly recognised as a powerful approach for capturing protein flexibility. Compared to traditional single-structure docking, ensemble-based methods enhance hit identification by accounting for conformational variability in viral proteins. Through the use of a global network of volunteer computers to perform these large-scale calculations, OpenZika aims to accelerate the discovery of new therapeutics for ZIKV infection. The docking results are made publicly available, enabling researchers to use the data to focus on compounds with the highest potential for therapeutic development. Docking calculations have been performed for the main proteins of the virus, including NS1, NS2B–NS3 protease, NS3 helicase, and NS5 polymerase. Since the project's inception in 2016, approximately four million docking jobs have been submitted to the World Community Grid, generating around three billion results. A number of the virtually chosen hits were subsequently selected for experimental validation.^15,67^ Another successful application of computational approaches for ZIKV drug discovery was reported by Abrams and co-workers, who combined quantitative high-throughput screening (qHTS) with an artificial intelligence (AI)-based QSAR model to identify inhibitors of ZIKV protease (ZVpro). The model was built using quantitative neighbourhoods of atom descriptors coupled with deep-learning techniques and subsequently applied in the virtual screening of over 100 000 compounds to enhance the identification of active molecules and accelerate the screening process. Selected hits were then validated using a series of orthogonal cell-based infection assays. Among these, two compounds, MK-591 and JNJ-40418677, emerged as the most potent, exhibiting anti-ZIKV activity by inhibiting ZVpro. Both compounds also effectively inhibited infection in neural stem cells. Interestingly, several antibiotics from the tetracycline family demonstrated stronger inhibition of ZIKV infection than the protease-targeted compounds. Notably, methacycline showed activity against ZIKV strains associated with neurological complications. It reduced viral load in the brain, limited the extent of infection, and alleviated the severity of virus-induced motor deficits in an immunocompetent animal model.^68^

**Fig. 8:**
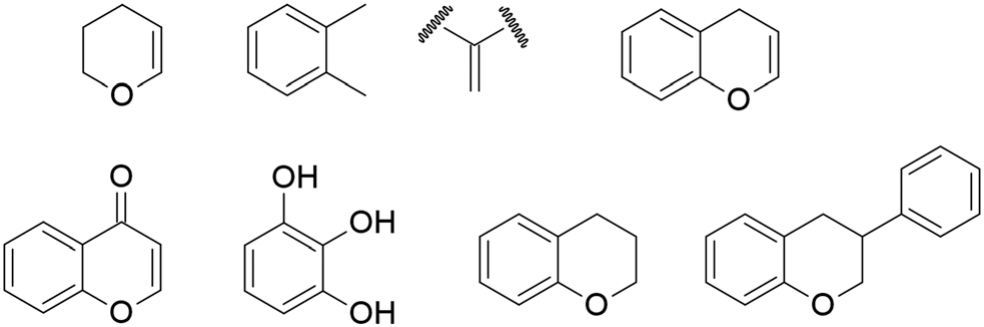
Structural features for the promoters of Zika virus (ZIKV) inhibitory activity.

**Fig. 9:**
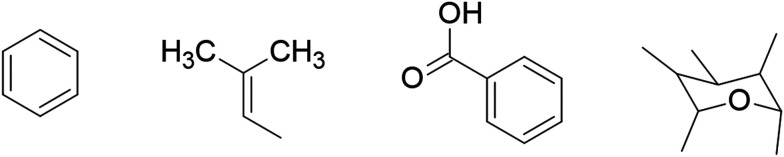
Structural features for the hinderers of Zika virus (ZIKV) inhibitory activity.

Another application of QSAR in the discovery of anti-ZIKV drug candidates was reported by Bhargava *et al.*, who aimed to identify the structural requirements of flavonoids for inhibiting ZIKV activity using Monte Carlo simulation-based QSAR and molecular docking. In their study, the optimal QSAR model was developed by combining simplified molecular input line entry system (SMILES) and hydrogen suppressed graph (HSG) descriptors with extended connectivity (EC1), achieving high predictive performance with statistical parameters of *R*^2^ = 0.9569 and *Q*^2^ = 0.9050. This model was then used to predict the inhibitory activity of flavonoids, indicating that the structural features shown in Fig. 8 are essential for enhancing anti-ZIKV activity, whereas the features illustrated in Fig. 9 act as hindrances. Based on these predictions, four flavonoids (amentoflavone, fisetin, isorhamnetin, and theaflavin-3-gallate) demonstrated higher inhibitory activity against ZIKV due to the presence of the structural features that promote the inhibitory activity in their molecules. Additionally, a docking study was performed to validate these findings, where the results showed that all four flavonoids exhibited higher binding affinities than the co-crystallised ligand.^69^

**Fig. 10:**
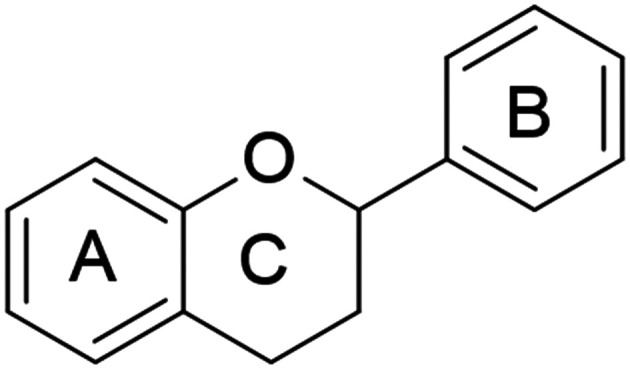
Basic structure of flavonoids.

A broad body of literature suggests that flavonoids represent a promising source for the development of ZVpro inhibitors. The ZVpro inhibitory activity primarily relies on intermolecular interactions between the active site of the viral protein and the molecule, including hydrophobic, hydrophilic, and electrostatic forces. The activity of flavonoids is largely influenced by the phenol group, which is highly acidic and can act as a nucleophilic species in the presence of weak bases. This property allows the insertion of various functional groups, such as alkyl, alkynyl, and triazole moieties. Flavonoid rings can also accommodate multiple substituents, including methyl, methoxy, hydroxy, and benzyl groups. Structural modifications of flavonoids can therefore be employed to enhance antiviral activity, reduce toxicity, and improve the half-life of the compounds. Lim and colleagues reported that both the number and position of the hydroxyl groups in the flavonoid structures favour inhibitory effects on ZVpro, whereas methoxy, prenyl, and glycosylation groups can hinder the anti-ZIKV activity. The presence of oxygen within a ring is essential for inhibitory activity, while glycone moieties are generally detrimental.^70,71^ Similarly, Zou *et al.* highlighted the role of hydroxyl groups in the B-ring and their importance for the anti-ZIKV activity of flavonoids (Fig. 10).^72^ Structural modifications can also improve flavonoids' bioavailability. For example: 1) introducing a nonpolar group like a hydrocarbon chain can increase lipid solubility, *e.g.*, etherifying the A-ring with an alkyl chain or methoxy group; 2) increasing polyhydroxylated groups enhances aqueous solubility by forming hydrogen bonds with water in biological environments.^73^

## Dengue virus (DENV)

Dengue virus (DENV) comprises four distinct serotypes, namely DENV-1, DENV-2, DENV-3, and DENV-4, which show antigenic variations in their envelope protein. Each serotype comprises multiple subtypes, resulting from genomic modifications, primarily detected across India. In addition to these, a fifth serotype (DENV-5) was identified in Malaysia in 2013. The serotype was detected in the patient's blood through viral isolation and genetic sequence analysis.

DENV is considered one of the leading causes of substantial morbidity and economic burden in many tropical and subtropical regions, particularly in Southeast Asia and the Indian subcontinent. The incubation period of DENV infection typically ranges from four to seven days. Clinical manifestations vary from asymptomatic or mild fever to severe forms, such as dengue haemorrhagic fever (DHF) or dengue shock syndrome (DSS), which are characterised by thrombocytopenia, coagulation abnormalities, leucopenia, plasma leakage, and increased capillary permeability. Fluid loss due to increased vascular permeability can lead to hypovolemic shock and multi-organ failure. Infection with one DENV serotype provides lifelong immunity against re-infection with the same serotype, but provides only temporary and partial protection against others. This limited cross-immunity may result in more severe disease upon secondary infection with a different serotype, primarily due to heterotypic immune responses and antibody-dependent enhancement (ADE). The first licensed dengue vaccine, tetravalent CYD (Dengvaxia), has not been approved for use in all countries. Major challenges in dengue vaccine development include the lack of appropriate animal models, incomplete understanding of disease pathogenesis, and the potential risk of ADE.^74,75^

## Inhibitors of DENV

The NS5 protein plays a central role in DENV replication and is highly conserved across all four serotypes, making it an attractive target for antiviral drug development. Consequently, several recent *in silico* studies have focused on identifying plant-derived natural compounds capable of inhibiting key functional domains of NS5, such as RdRp and MTase, as well as other essential NS proteins, including the NS2B/NS3 protease.

Jarerattanachat and colleagues screened 91 natural compounds derived from ginseng and notoginseng against the NS5 MTase domain using molecular docking. Isoquercitrin emerged as a promising hit and was further validated through 500 ns molecular dynamics (MD) simulations and molecular mechanics with Poisson–Boltzmann surface area (generalized born) surface area MM–PB(GB)SA binding energy calculations.^76^ The compound demonstrated stable binding at both the AdoMet binding site and the RNA capping site, along with favourable absorption, distribution, metabolism, and excretion (ADME) properties, and low cytotoxicity (CC_50_ > 20 μM), confirming its potential as a viable anti-DENV candidate.^77^

Building upon this approach, Halder *et al.* conducted a virtual screening of 922 phytochemical compounds against the DENV-2 2′O-MTase, a key enzyme involved in viral RNA capping and immune evasion. Their workflow combined drug-likeness evaluation using SwissADME, absorption, distribution, metabolism, excretion, and toxicity (ADMET) profiling *via* pkCSM, molecular docking using AutoDock Vina and SwissDock, and MD simulations with Desmond. Out of the screened compounds, 45 showed promising activity, with CHEMBL376820 emerging as the most potent candidate.^78^ On the other hand, Roney *et al.* focused on targeting another domain of NS5 using a combination of ligand- and structure-based approaches to screen 42 natural antiviral compounds against the RdRp active site. Following Lipinski's rule of five and ADMET filtering, 3′-*O*-methyldiplacol was identified as a strong binder to the RdRp active site, with a favourable docking score and stable binding interactions.^79^

Beyond NS5, Bhattarai and co-workers extended these computational efforts to assess the inhibitory potential of 31 secondary metabolites derived from medicinal plants against both NS2B/NS3 protease and NS5 polymerase. Their docking and pharmacokinetic analyses identified agathisflavone and pectolinarin as lead inhibitors of NS2B/NS3 and NS5, respectively. Additional promising compounds included epigallocatechin gallate, pinostrobin, and acacetin-7-*O*-rutinoside. Notably, agathisflavone and pectolinarin exhibited higher LD_50_ values (1430 and 5000 mg kg^−1^, respectively) than reference antivirals, which suggests a favourable safety profile.^80^

Complementing these docking-based approaches, QSAR methodologies have also been applied to identify potential NS2B/NS3 protease inhibitors. Mirza *et al.* employed an integrated strategy combining QSAR, pharmacophore modelling, and molecular docking to screen compounds from the ZINC database as potential inhibitors of the NS2B/NS3 protease.^81^ The top-ranked compounds displayed strong predicted binding affinities with the catalytic triad residues (His51, Asp75, and Ser135), suggesting effective inhibition of the enzyme. Subsequently, the same group extended their work using MD simulations to further characterise the stability and interaction dynamics of the protein–ligand complexes.^82^ Moreover, structure-based pharmacophore modelling (E-pharmacophore) coupled with molecular fingerprint-based virtual screening of the Otava compound database led to the identification of additional promising hits against the DENV NS2B/NS3 protease.^83–85^ Similarly, another study developed a ligand-based pharmacophore model and used ZINCPharmer to screen compounds from the ZINC database.^86^ A 2D-QSAR model was developed and validated using reported 4-benzyloxy phenyl glycine derivatives and was utilised to predict the IC_50_ values of unknown compounds. Further, two hits, ZINC36596404 and ZINC22973642, exhibited high predicted pIC_50_ values (6.477 and 7.872) and favourable docking scores (−8.3 and −8.1 kcal mol^−1^), with good stability confirmed *via* MD and MM-PBSA energy calculations.^83^ In another QSAR study, a multiple linear regression model with genetic function approximation (MLR-GFA) was used to evaluate phthalazinone derivatives. The model demonstrated high statistical robustness (*R*^2^ = 0.9555, *Q*^2^ = 0.9059) and was validated with docking studies against the DENV-2 NS2B/NS3 protease (PDB ID: 6MOL). Among the tested compounds, compound 21 showed the highest binding affinity (−8.9 kcal mol^−1^), correlating well with its pIC_50_ and confirming the predictive power of the QSAR model.^87^

**Fig. 11:**
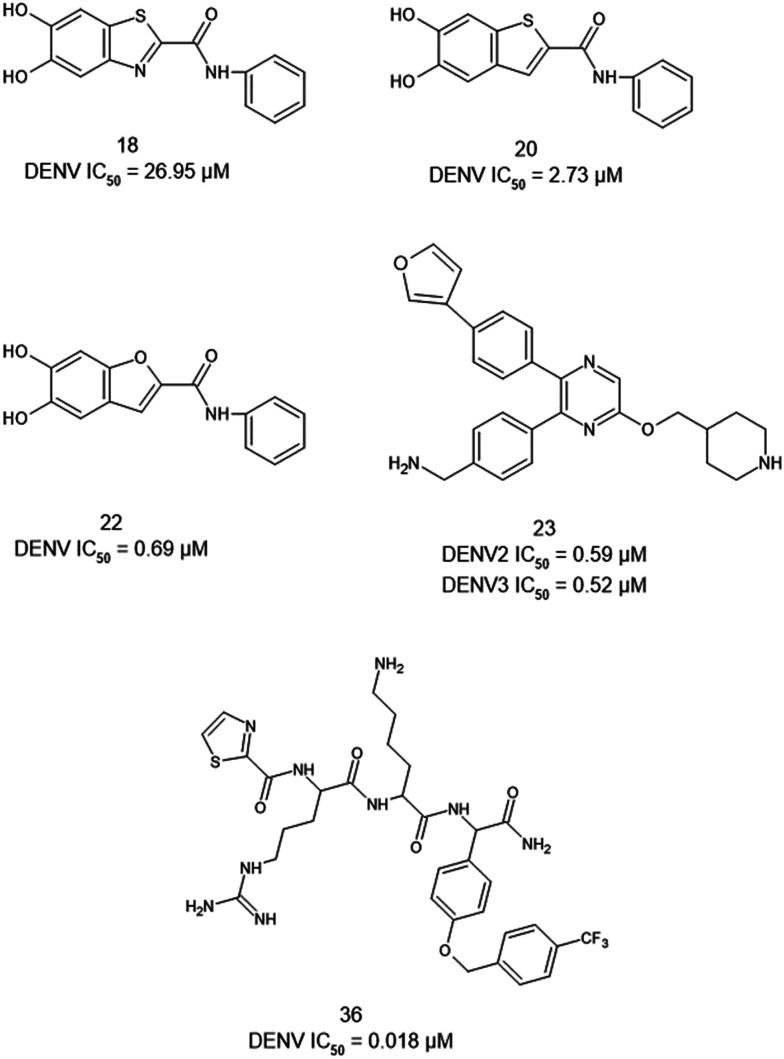
Structural features of representative DENV NS2B/NS3 protease inhibitors, including both allosteric and orthosteric compounds.

Inhibiting the dengue NS2B/NS3 protease requires careful consideration of the structural features that govern substrate recognition and catalytic activity. Effective inhibitors typically target the protease catalytic cavity or engage in precise interactions with the flexible NS2B cofactor, whose N-terminal region is essential for forming the active protease. Compounds can either stabilise inactive conformations of the protease or disrupt the conformational changes required for catalysis. Key substrate-binding pockets, such as P1 and P3′, are critical for activity, and mutations in conserved residues within the active site significantly reduce enzymatic function, underscoring their importance for catalysis. Certain N-heterocyclic scaffolds, including benzofuran and pyrazine rings, have emerged as promising scaffolds for broad-spectrum NS2B/NS3 inhibitors. Structural modifications can significantly influence antiviral activity. Certain N-heterocycles, such as benzofuran and pyrazine rings, are promising scaffolds for broad-spectrum NS2B/NS3 inhibitors. For instance, replacing a benzothiazole ring with a benzofuran nucleus generated some of the most potent inhibitors, whereas substitution with a benzothiophene improved activity to a lesser extent (Fig. 11, compounds 18, 20, and 22). Hydrophilic substituents, such as hydroxyl groups, enable hydrogen bonding with active-site residues, while additional modifications, like the 4-(furan-3-yl) phenyl group, further enhance inhibitory potency. A notable example is the allosteric inhibitor (Fig. 11, compound 23), which features a pyrazine ring connected *via* an ether bridge to a piperidine ring, with substituents at positions 5 and 6, including a 4-phenylmethylamino and a 4-(furan-3-yl) phenyl group. This structurally unique compound effectively inhibits DENV serotypes 2 and 3, with IC_50_ values of 0.59 μM and 0.52 μM, respectively, demonstrating its broad-spectrum potential.^88^

Further, orthosteric inhibitors have also shown promise, particularly thiazolidinylcarbonyl-Arg-Lys-(OCH_2_C_6_H_6_(4-CF_3_)-Phg-NH_2_) (Fig. 11, compound 36), which exhibited an IC_50_ of 18 nM against DENV2. This compound interacts directly with the catalytic triad residues His51, Asp75, and Ser135, effectively blocking enzymatic activity. Collectively, these findings emphasise the importance of combining structural insights with strategic chemical modifications to develop potent and selective NS2B/NS3 protease inhibitors.^89^

## West Nile (WNV)

West Nile virus (WNV) was first isolated in 1937 from the blood of a symptomatic patient in Uganda.^90^ Since its discovery, WNV has evolved into a major public health concern, now considered the most geographically widespread member of the flavivirus genus. The virus comprises nine distinct genetic lineages, among which lineages 1, 2, and, more recently, 3 are known to cause diseases in humans.^91^ Lineage 1 is the most widely distributed and is responsible for most outbreaks worldwide, while lineage 2, initially restricted to sub-Saharan Africa, has expanded into Europe over the past two decades, leading to increasing neuroinvasive cases.^92^ WNV is currently endemic in Africa, the Middle East, Europe, Asia, Oceania, and the Americas. Since its introduction into North America in 1999, the virus has spread rapidly across the United States, Canada, Mexico, and parts of South America.^93^ Major outbreaks have been reported in the United States (2021), Tunisia (2018 and 2023), and Italy (2022 and 2024).^94–97^ The virus has also been detected in migratory bird populations, which suggests long-distance avian-mediated transmission between continents.

The primary vectors for WNV transmission are mosquitoes of the Culex genus. Other mosquito species, such as *Aedes albopictus* and *Aedes aegypti*, have been shown to support infection under experimental conditions, which indicates possible vector expansion in warmer climates. Birds serve as the principal reservoirs, maintaining the enzootic cycle, whereas humans and equids represent dead-end hosts because they do not develop sufficient viremia to perpetuate transmission. Approximately 25% of infected individuals develop a symptomatic infection and experience West Nile fever, which is characterised by mild to moderate symptoms including fever, headache, muscle pain and fatigue. Around 1% of infections progress to West Nile neuroinvasive disease, manifesting as encephalitis or acute flaccid paralysis, particularly in elderly or immunocompromised individuals. The case-fatality rate for neuroinvasive disease ranges between 4% and 14%.^98^

Several studies have highlighted the significant impact of climate change on the global expansion of WNV. Warmer temperatures and altered precipitation patterns enhance mosquito breeding, shorten viral incubation periods within vectors, and facilitate transmission to new geographic regions. Consequently, the frequency and geographic range of WNV outbreaks are projected to increase, particularly in temperate areas previously considered low-risk.^93,99^ Currently, there are four licensed vaccines available for veterinary use in horses; however, no vaccine or antiviral treatment has been approved for human use. Supportive therapy remains the mainstay of clinical management. Ongoing research focuses on the development of human vaccines using recombinant viral vectors, mRNA-based platforms, and monoclonal antibodies targeting the viral E protein, which mediates host cell entry and immune recognition.^100^

## Inhibitors of WNV

Previous studies have reported significant biological activities of dispiropyrrolidines, including antitumor, antineoplastic, antimicrobial, antifungal, antimycobacterial, and antidiabetic effects.^101–106^ Accordingly, Ali *et al.* synthesised a series of 14-dibenzylidene-1-phenylethylpiperidine-4-ones and evaluated their antimycobacterial activity against *Mycobacterium tuberculosis*, where compound 3d ((3*E*,5*E*)-3,5-bis (4-triflouromethylbenzyledene)-1-phenethylpiperidin-4-one) was found to be the most active with a minimum inhibitory concentration (MIC) of 27.00 μM.^107^ Building on their previous achievement and extending this research domain, their subsequent work focused on the design of a new series of spiropyrrolidine derivatives as potential WNV NS2B–NS3 protease inhibitors, synthesised *via* a [3+2]-cycloaddition reaction involving isatins, sarcosine, and (*E*)-3,5-bis(arylidene)-4-piperidones. The chemical structures of all synthesised compounds were comprehensively characterised using Fourier transform infrared (FT-IR), 1D- and 2D-nuclear magnetic resonance (NMR), and high-resolution mass spectrometry (HRMS) analyses. Molecular docking studies using AutoDock 4.2 were conducted to evaluate the interactions of the spiropyrrolidines with NS2B–NS3 protease and to provide insights into potential structural optimisations. Among the synthesised derivatives, compound 5c exhibited the most favourable binding energy (−7.71 kcal mol^−1^) and an estimated inhibition constant (Ki) of 1.73 μM. The compound formed two hydrogen bonds with Asn84 *via* the C=O and –NH groups of its oxindole ring, in addition to van der Waals interactions with Gly1151 and Gly1153, and π–π stacking with Tyr1161, which altogether stabilised the ligand within the protease active site and highlighted its potential as a WNV NS2B–NS3 inhibitor.^108^

**Fig. 12:**
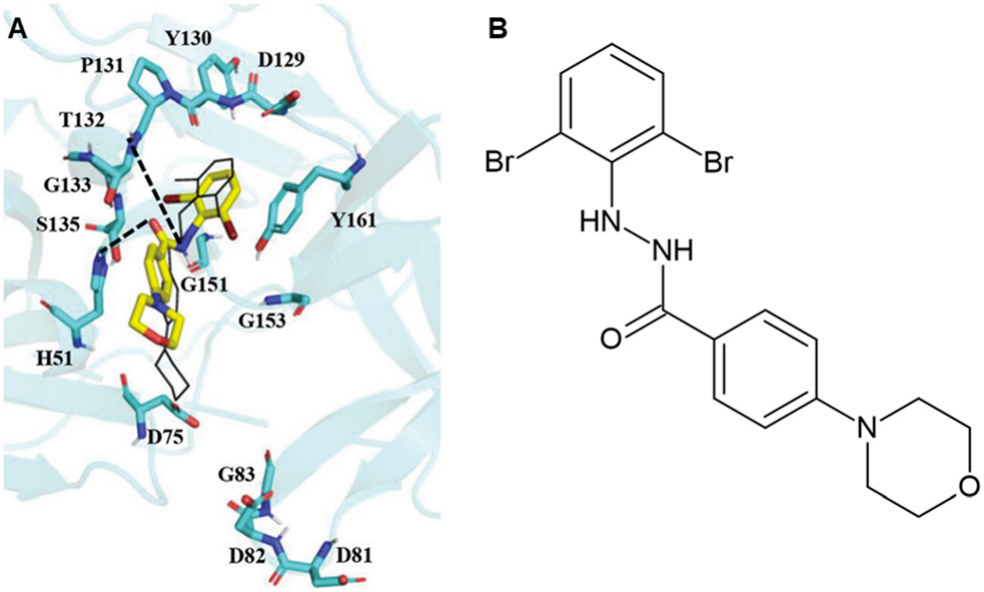
A) Docking pose of compound 11q in the binding site of the WNV-NS2B–NS3. B) The 2D molecular structure of 11q. Reproduced from Yadav R. Gene & protein in disease. 2025; under the terms of the Creative Commons CC BY license.^110^

Similarly, a recent study conducted by Yadav and colleagues targeted the NS2B–NS3 protease across WNV and DENV, investigating the synthetic aryl benzoyl hydrazide derivative *N*-(((2,6-dibromophenyl)amino) methyl)-4-morpholinobenzamide (11q). Previously, 11q was shown to inhibit the influenza virus, exhibiting nanomolar antiviral activity against H1N1 and Flu B viruses by targeting RdRp.^109^ It also bound strongly to the ZIKV NS2B–NS3 protease, outperforming SYC-1307, a known ZIKV protease inhibitor.^88^ Given the high structural and sequence conservation of NS2B–NS3 among ZIKV, DENV, and WNV, the binding potential of 11q was assessed against the DENV and WNV proteases. Molecular docking, MD simulations, and binding free energy calculations revealed that 11q binds strongly to DENV and WNV NS2B–NS3 proteases, with binding free energies of −15.80 ± 3.34 kcal mol^−1^ and −13.13 ± 2.56 kcal mol^−1^, respectively. The slightly more favourable binding to DENV is consistent with the observed ZIKV interactions. Further, the results of 100 ns MD simulations demonstrated that the WNV-NS2B–NS3-11q complex remained stable, with a root mean square deviation (RMSD) value below 2.5 Å and radius of gyration (*R*_g_) ranging between 15.5 and 16.5 Å. Throughout the simulation, 11q maintained a conformation closely resembling its initial docked pose. The molecular interactions that facilitated the ligand's stabilisation within WNV NS2B–NS3 included π–π interactions with Tyr161, which contributed most to binding affinity, as well as weak hydrogen bonds with Thr132 and Ser135, which are important to maintain the ligand's orientation within the binding site (Fig. 12). Importantly, 11q also demonstrated stable binding to DENV and ZIKV proteases; hence, the authors suggested that this molecule might serve as a pan-antiviral NS2B–NS3 inhibitor across different flavivirus species.^110^

**Fig. 13:**
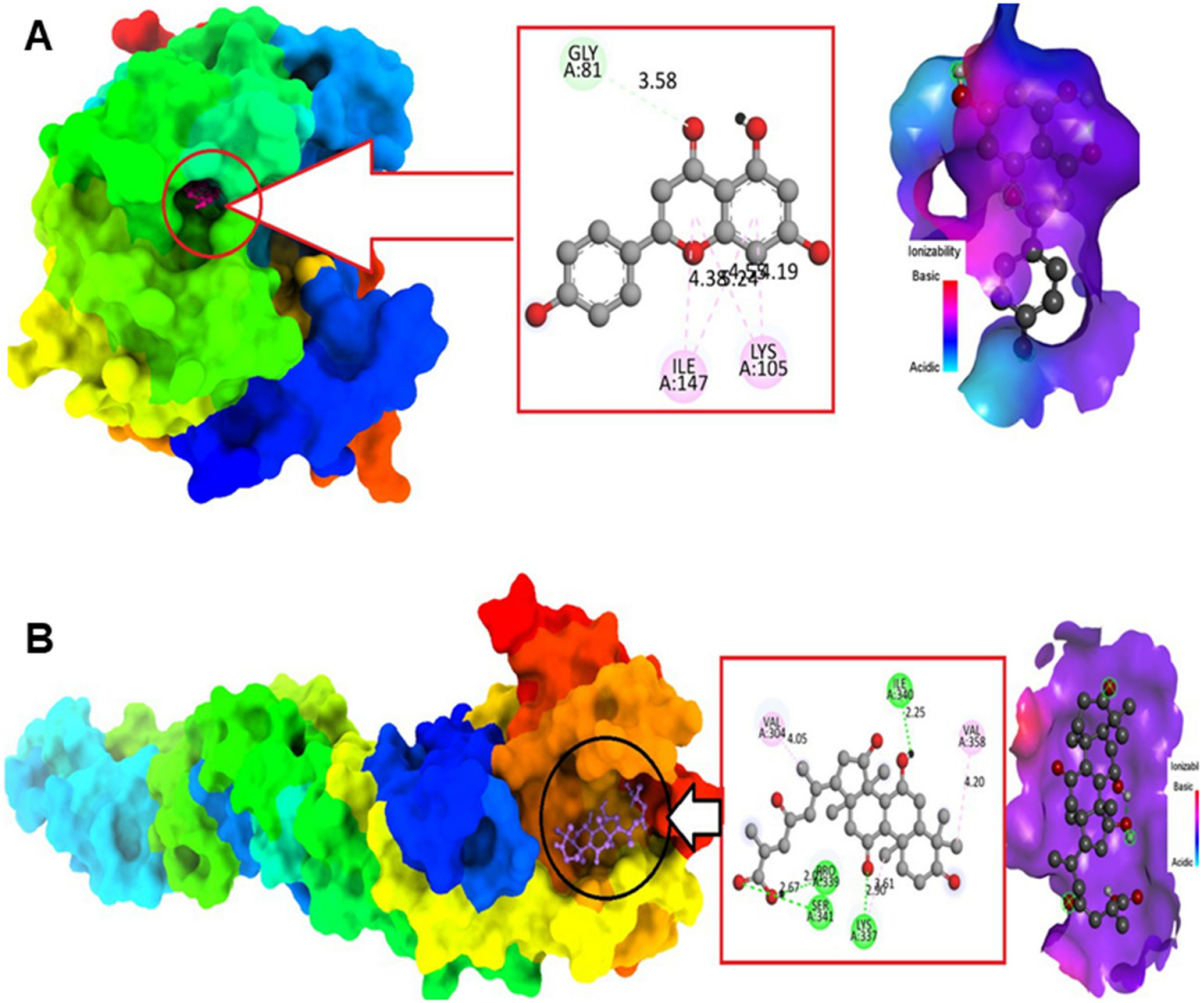
Interactions of protein–ligand complexes. A) Apigenin bound to West Nile virus (WNV) methyltransferase (MTase). B) Ganoderic acid bound to WNV envelope (E) glycoprotein. Reproduced from Akash S. Frontiers in Microbiology. 2023; under the terms of the Creative Commons CC BY license.^111^

In addition to synthetic inhibitors targeting the NS2B–NS3 protease, natural products have also emerged as promising candidates against WNV, particularly by interacting with the E glycoprotein and NS5 MTase. Akash and co-workers conducted a comprehensive literature review to select twelve natural compounds with reported antiviral activity, including apigenin, resveratrol, hesperetin, fungisterol, lucidone, ganoderic acid, curcumin, kaempferol, cholic acid, chlorogenic acid, pinocembrin, and sanguinarine. Molecular docking analyses were performed using BIOVIA Discovery Studio v16.1.0 to evaluate the binding affinities of these compounds to WNV NS5 MTase and E glycoprotein. Among the tested molecules, apigenin and curcumin exhibited the highest docking scores against NS5 MTase (−8.5 and −8.3 kcal mol^−1^, respectively), whereas ganoderic acid and cholic acid showed the strongest binding to the E glycoprotein (−8.0 and −7.6 kcal mol^−1^, respectively). Apigenin interacted with the MTase residues Val132, Asp131, Phe133, Lys105, Asp146, Trp87, Gly58, and Asp79, while curcumin interacted with Lys105 and Ile147. On the other hand, ganoderic acid formed contacts with Pro339, Val304, Lys337, Arg338, Val385, Val343, and Asn347, and cholic acid bound to Leu340, Val358, Val304, Lys337, Ser341, and Pro339 of E glycoprotein. To further evaluate the stability and dynamic behaviour of the top-performing compounds identified in the docking experiments, MD simulations were conducted over a 100 ns timeframe. Analysis of the RMSD revealed that the complexes formed between curcumin or ganoderic acid and the WNV NS5 MTase or E glycoprotein maintained stable conformations throughout the simulation period, suggesting a high degree of structural integrity in the ligand-protein interactions. In contrast, the apigenin complexes exhibited greater fluctuations in RMSD, indicating relatively less stable binding under dynamic conditions. Complementary, the root mean square fluctuation (RMSF) analysis demonstrated that curcumin and ganoderic acid induced reduced flexibility in key binding regions of the proteins, further supporting the notion of more rigid and stable interactions with their respective targets. Interestingly, despite the apparent stability of the ganoderic acid-E glycoprotein complex, the calculated binding free energy (Δ*G*) was 2.99 kcal mol^−1^, suggesting that this compound may actually possess the weakest thermodynamic binding among the tested molecules, highlighting a possible discrepancy between structural stability and binding affinity. Notably, of all the shortlisted compounds, only apigenin complied with Lipinski's rule of five, emphasising its favourable physicochemical properties and potential as a drug-like antiviral candidate (Fig. 13).^111^

**Fig. 14:**
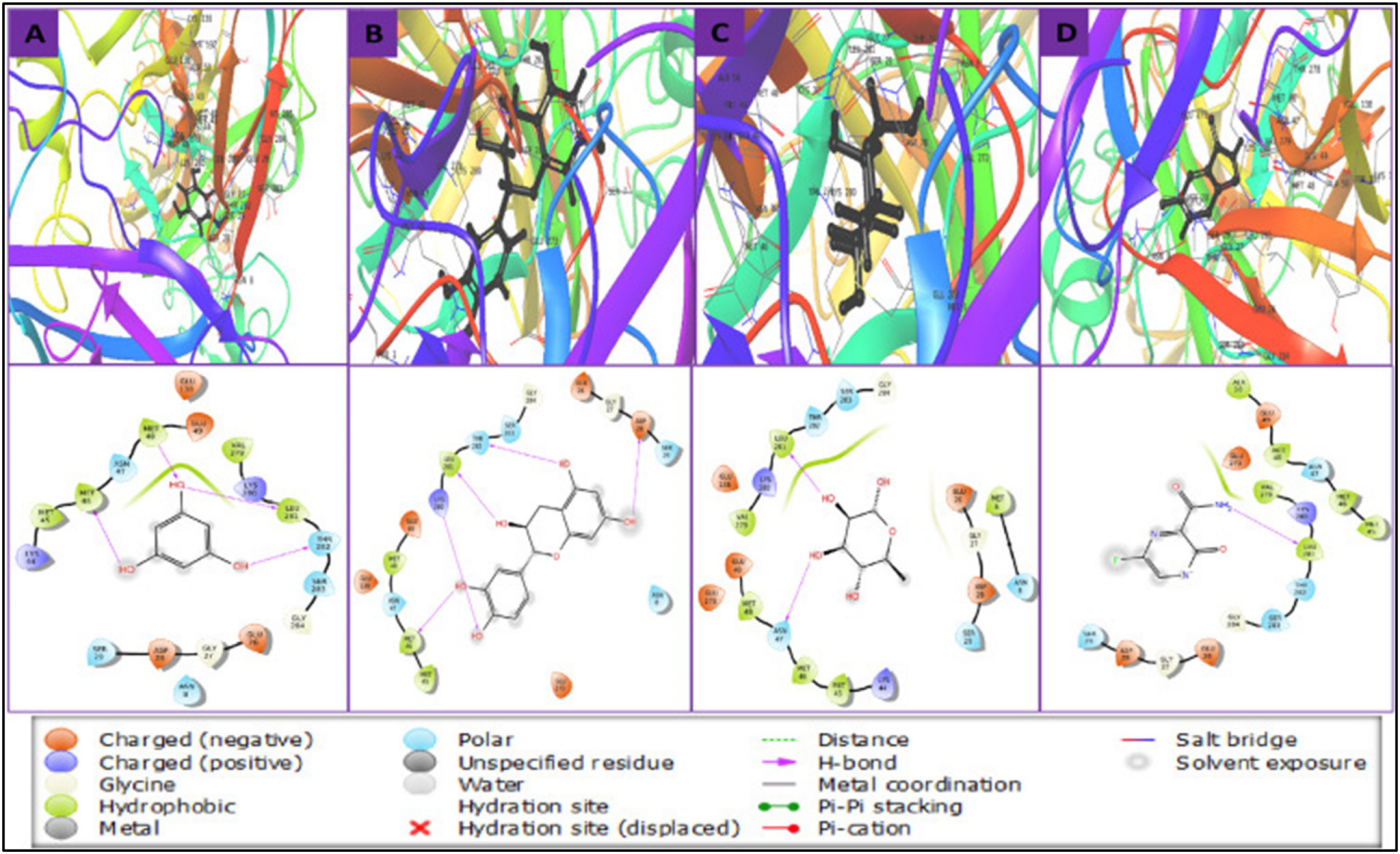
Binding interactions between the WNV's envelope (E) glycoprotein and the top hits in 3D and 2D formats. A) Phloroglucinol. B) Cianidanol. C) L-Rhamnose. And D) Favipiravir. Reproduced from Siddiquee NH. PLoS One. 2025; under the terms of the Creative Commons CC BY license.^112^

Building on this structure-based approach, a subsequent study conducted a large-scale virtual screening of 5375 phytochemicals retrieved from the Indian Medicinal Plants, Phytochemistry and Therapeutics (IMPPAT) database, specifically targeting the E glycoprotein of the WNV. In this study, blind docking was performed using the Glide v8.8 module integrated within Schrödinger Maestro v12.5.139, and the clinically known antiviral drug favipiravir was included as a reference compound to benchmark docking performance. Among the screened compounds, several phytochemicals demonstrated strong binding potential toward the E glycoprotein. The top-ranked ligands were phloroglucinol (derived from *Phyllanthus emblica*), cianidanol (from *Tamarindus indica*), and L-rhamnose (from *Plantago ovata*), with docking scores of −7.46, −5.80, and −5.61 kcal mol^−1^, respectively. Subsequent MM-GBSA calculations provided further insights into the thermodynamic stability of the docked complexes, yielding estimated binding free energies of −29.16, −33.45, and −32.02 kcal mol^−1^ for the same ligands. Detailed interaction analysis revealed that all three compounds, along with favipiravir, formed hydrogen bonds and other non-covalent interactions with key residues located within the active binding pocket of the E protein, including Leu281, Thr282, Ser283, and Ser89. Additional hydrophobic contacts with Met45, Met46, and Met48 further stabilised the ligand–protein interactions (Fig. 14). MD simulations conducted over 100 ns confirmed the conformational stability of these complexes, with cianidanol exhibiting the most consistent and tightly bound interaction profile throughout the simulation period. Collectively, these findings highlight the potential of naturally derived compounds, particularly cianidanol, as promising inhibitors of the WNV E glycoprotein. Their superior binding affinities compared to favipiravir suggest that these phytochemicals may interfere with viral attachment and fusion processes, thereby offering a basis for the development of novel antiviral agents targeting flavivirus entry mechanisms.^112^

The objective of this pipeline was to construct a machine learning-guided automated quantitative structure–activity relationship (AutoQSAR) model capable of performing *in silico* prediction and generation of potential antiviral leads against DENV and WNV, with a specific focus on their MTase enzymes. Within this integrated framework, the AutoQSAR model was first trained on curated subsets of known active and inactive compounds, identifying key molecular descriptors that influence antiviral activity. Using these learned relationships, the system automatically generated novel small-molecule structures predicted to exhibit high binding affinity toward the MTase active site. The workflow then employed AutoDock Vina to perform automated molecular docking simulations for each of the generated compounds, assessing their interaction patterns, binding conformations, and calculated free energies of binding to the viral protein target. Following docking, the top-ranked hits were filtered through drug-likeness criteria using Lipinski's rule of five to evaluate their pharmacokinetic suitability for oral administration. This automated selection ensured that only compounds with favourable molecular weight, polarity, hydrogen-bonding potential, and lipophilicity were retained as potential candidates. Among the screened molecules targeting the WNV MTase, one compound, PubChem CID439610, was identified as particularly promising. It exhibited a calculated binding energy of −7.4 kcal mol^−1^ and demonstrated stable interactions with several catalytically important residues, including Glu218, Gly81, Lys182, Asp146, Glu111, Cys82, Ser56, and Gly58, suggesting a strong and specific affinity for the active site of the viral enzyme. An important feature of this automated platform is its direct linkage to the continually expanding PubChem database, which allows the program to dynamically mine new chemical data each time it is executed. This ensures that the pool of candidate molecules reflects the most up-to-date chemical space available, facilitating rapid and adaptive drug lead generation. By integrating data mining, machine learning-based molecular design, and automated docking into a single framework, this system represents a significant advancement toward fully automated *in silico* drug discovery for flaviviral MTase inhibitors.^113^

## Japanese encephalitis virus (JEV)

Japanese Encephalitis virus (JEV) is one of the leading causes of viral encephalitis in humans across Eastern and Southeastern Asia. It is a zoonotic, mosquito-borne flavivirus comprising five genotypes (I–V) and is transmitted to humans through the bite of an infected *Culex tritaeniorhynchus* mosquito.^114,115^ Wild wading birds constitute the primary natural reservoirs, while pigs serve as the main amplifying hosts and are strongly implicated in human outbreaks.^116^ In contrast, humans are considered dead-end hosts, as their viraemia levels are insufficient to enable transmission back to the mosquito vector.^117^ In most individuals, JEV infection causes only a mild febrile illness; however, approximately 1% of cases progress to Japanese encephalitis, which has a mortality rate of up to 30%. Among survivors, 25–30% suffer from long-term neurological sequelae, including lifelong disabilities and cognitive impairments.^6,118^ As a neuroinvasive virus, JEV can cross the blood–brain barrier (BBB) and access the central nervous system (CNS) through several mechanisms, leading to acute encephalitis.^119^ These include: 1) transcellular transport across endothelial cells *via* transcytosis or direct infection and subsequent release of viral particles into the CNS; 2) entry within infected leukocytes, particularly monocytes, through a Trojan Horse mechanism; 3) paracellular passage across regions where tight junctions are disrupted, increasing vascular permeability; 4) retrograde neuronal transport following infection of peripheral neurons that project to the CNS; and 5) direct inoculation into the cerebrospinal fluid (CSF) at sites lacking a functional BBB, such as the circumventricular organs. The clinical course of Japanese encephalitis typically begins with nonspecific symptoms such as headache, diarrhoea, coryza, and rigours, which may progress to altered consciousness and seizures. Severe complications of the disease include movement disorders, flaccid paralysis, and deafness.^114^

Currently, no antiviral treatments are available for Japanese encephalitis because of the incomplete understanding of the mechanisms underlying viral neuropathogenesis. However, widespread immunisation and eradication programs have successfully reduced or nearly eliminated the disease in economically developed Asian countries such as Taiwan, Korea, and Japan. Despite these achievements, the geographical range of JEV continues to expand across Southeast Asia, even in regions with ongoing vaccination programs. This expansion is driven by the increasing distribution of mosquito vectors and limited surveillance, coupled with a poor understanding of transmission dynamics.^118^ Furthermore, global climate change is expected to exacerbate the situation by expanding mosquito habitats, thereby facilitating the introduction of JEV into previously non-endemic areas.

## Inhibitors of JEV

**Fig. 15:**
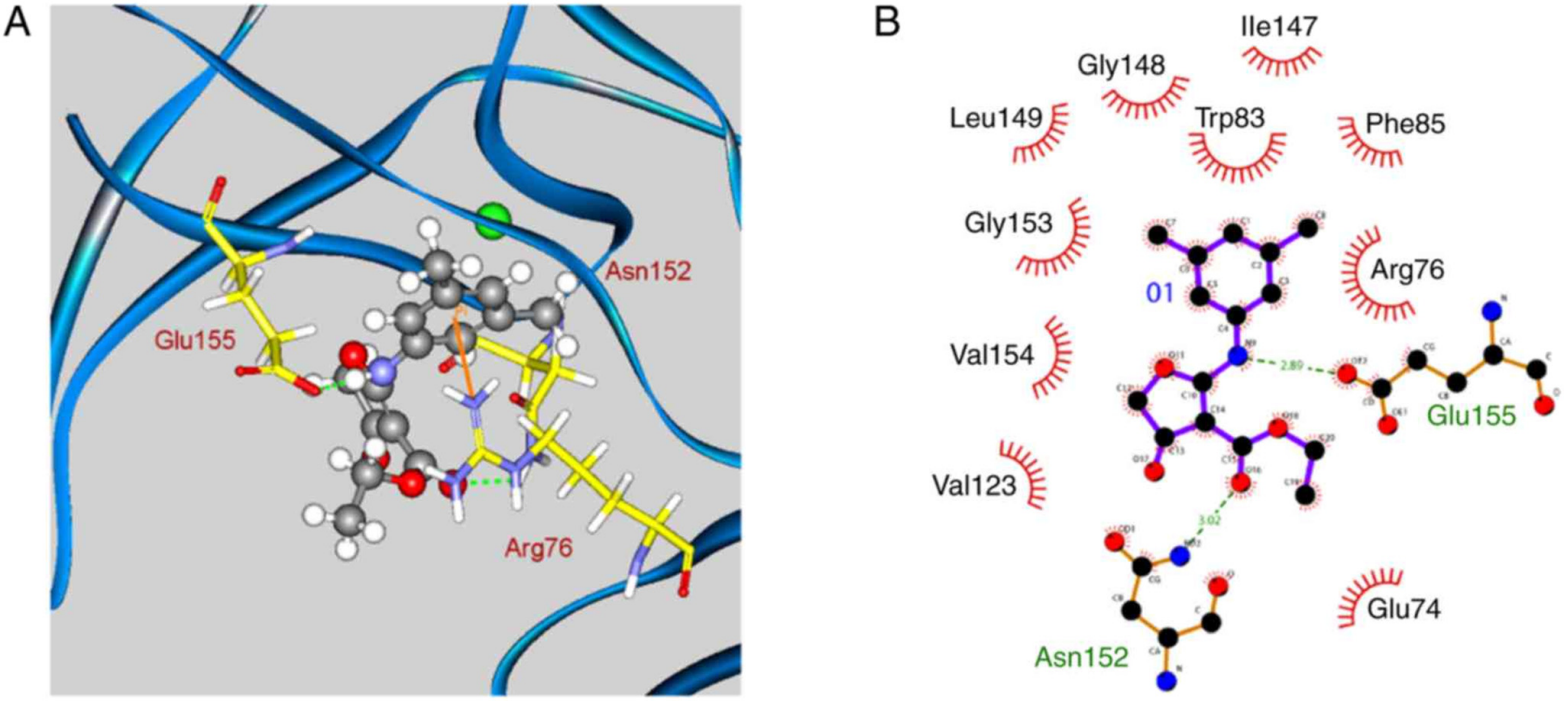
Docking pose of compound CW-33 in the binding site of the JEV protease. A) 3D and B) 2D protein–ligand interactions. Reproduced from Chen K-C *et al.* International Journal of Molecular Medicine 2019; under the terms of the Creative Commons CC BY license.^120^

Natural compounds have been extensively investigated for their antiviral activity against JEV, with several studies demonstrating their ability to inhibit viral replication and infection. Among these, furoquinoline alkaloids are of particular interest due to their broad pharmacological activities. CW-33 (ethyl 2-(3′,5′-dimethylanilino)-4-oxo-4,5-dihydrofuran-3-carboxylate), a synthetic derivative of furoquinoline alkaloids, was identified by Chen *et al.* as a promising antiviral candidate. The compound exhibited potent inhibitory effects on JEV replication by targeting the NS2B–NS3 serine protease. Further studies revealed that CW-33 and related furanonaphthoquinone derivatives inhibited both viral RNA and protein synthesis, thereby preventing JEV infection. Molecular docking studies elucidated critical protein–ligand interactions underlying CW-33 activity. The secondary amine group was identified as an essential pharmacophore, forming a hydrogen bond with Glu155, while the phenyl ring established π-cation interactions with Arg76. Additionally, the ethyl moiety engaged in hydrophobic contacts with Glu74, and hydrophobic substituents at the meta-position of the phenyl ring further enhanced antiviral efficacy (Fig. 15). The QSAR modelling supported these findings and highlighted the structural features required for optimal protease binding.^120^

Similarly, Chaudhury and co-workers investigated the anti-JEV potential of pokeweed using *in silico* approaches. The pokeweed antiviral protein (PAP), previously reported to inhibit viral RNA synthesis, was shown to bind with high affinity to the viral NS5 protein through protein–protein docking. In addition, esculentic acid from pokeweed demonstrated strong binding affinity toward both NS3 and NS5, suggesting a potential multi-target inhibitory effect. Computational assessments indicated that pokeweed's phytoconstituents are not pan-assay interference compounds (PAINs) and are less likely to cause undesirable effects. Although their water solubility was moderate, the compounds demonstrated favourable gastrointestinal absorption and acceptable partition coefficients (log *P* < 5), consistent with Lipinski's rule of five. Considering their potential for both direct and indirect interactions with JEV proteins, pokeweed mother tincture may represent a useful complementary therapy approach for JEV infection.^121^ Other studies have employed molecular docking and MD simulations to explore the inhibitory potential of phytoconstituents such as withaferin A (from *Withania somnifera*) and andrographolide (from *Andrographis paniculata*) against JEV. Andrographolide demonstrated strong binding affinity, particularly toward the NS3 protease, compared with the NS3 helicase and NS5 RdRp, whereas withaferin A exhibited favourable interactions with viral RdRp and NS5, suggesting its potential to interfere with multiple stages of viral replication. MD simulations further confirmed the stability of both compounds within the active sites of their respective binding pockets. In addition, both compounds showed favourable pharmacokinetic and safety profiles, adhering to drug-likeness parameters and exhibiting low predicted toxicity. Notably, andrographolide demonstrated *in vitro* inhibitory activity against the NS3 protease, while withaferin A exhibited additional immunomodulatory and cytotoxic properties that may enhance its antiviral effects. Collectively, these findings highlight the potential of plant-derived molecules as promising candidates for therapeutic development against JEV, although further preclinical and clinical validation remains necessary.^122,123^

**Fig. 16:**
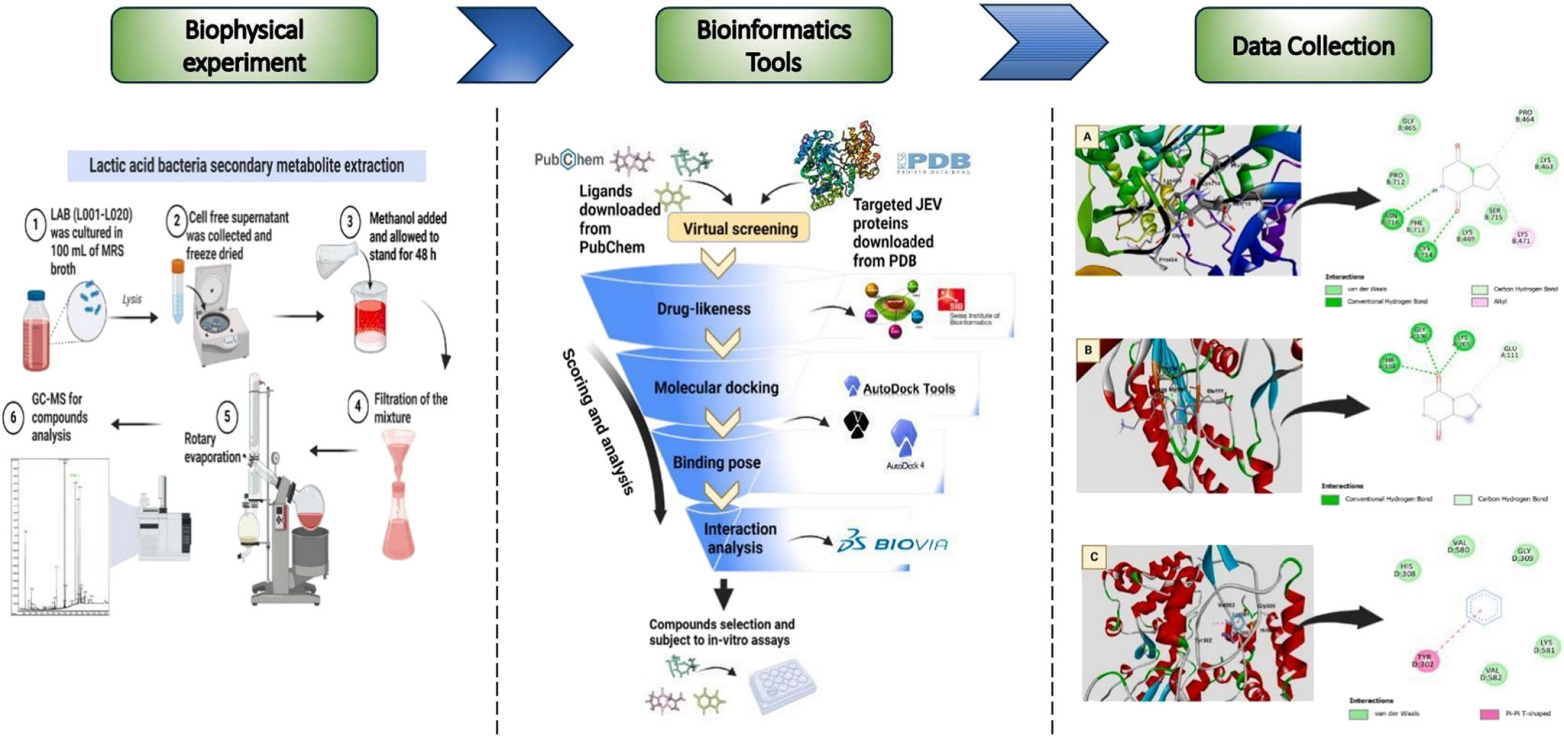
Screening workflow of lactic acid bacterial extracts for anti-JEV activity. Reproduced from Mansor *et al.* Results in Chemistry. 2025; under the terms of the Creative Commons CC BY license.^127^

Several *in silico* studies have also investigated lactic acid bacteria (LAB)-derived metabolites as potential inhibitors of JEV proteins. LAB are widely recognised for their probiotic effects; however, emerging evidence suggests that both LAB and their bacteriocins may possess antiviral properties against a range of pathogens, including enteroviruses, gastroenteritis viruses, and respiratory viruses.^124–126^ Three main antiviral mechanisms have been proposed: 1) direct interaction with viral particles to limit infectivity, 2) secretion of antiviral substances, and 3) modulation of host immune responses. Accordingly, Mansor *et al.* conducted a study in which fourteen compounds extracted from twenty LAB strains were screened using blind molecular docking with AutoDock Vina. Among these, three compounds, Comp-A (3-isobutyl-2,3,6,7,8,8a-hexahydropyrrolo[1,2]pyrazine-1,4-dione), Comp-B (pyrrolo[1,2-*a*]pyrazine-1,4-dione, hexahydro-3-(phenylmethyl)), and Comp-C (2,4-di-*tert*-butylphenol), exhibited the strongest binding affinities against JEV proteins, with docking scores of −7.3, −7.8, and −8.1 kcal mol^−1^, respectively. The predicted binding modes revealed stable interactions with key amino acid residues within the active pockets, supported by hydrogen bonding and hydrophobic contacts. Binding free energy calculations and MD simulations further confirmed the stability of these protein–ligand complexes. Moreover, the *in silico* pharmacological evaluation demonstrated that all three compounds possess favourable ADMET and drug-like properties, suggesting a promising safety and efficacy profile (Fig. 16).^127^

## Yellow fever (YFV)

Yellow Fever is an acute mosquito-borne viral haemorrhagic disease caused by the Yellow Fever virus (YFV). The virus primarily infects humans and non-human primates, with infected primates serving as natural reservoirs. YFV is transmitted to humans through the bite of infected *Aedes mosquitoes*, predominantly *Aedes aegypti* in urban settings. Clinically, yellow fever presents with a broad spectrum of manifestations, ranging from asymptomatic or mild febrile illness to severe, life-threatening disease. Common symptoms include fever, headache, myalgia, nausea, and vomiting, which may progress to jaundice, hepatomegaly, acute liver and kidney failure, and haemorrhage, which can rapidly lead to shock and death. Approximately 40% of symptomatic cases result in a fatal outcome.^128^

Evolutionary studies indicate that YFV originated in Africa, where it co-evolved with African primates and mosquitoes. The virus was likely introduced into the Americas and Europe during the colonial era through the transatlantic slave trade. Historically, Yellow Fever was one of the most feared infectious diseases between the 15th and 19th centuries due to its devastating outbreaks and high mortality rates.^129^ Despite limited understanding about the disease at that time, in the 19th century, physicians suspected that Yellow Fever was not transmitted directly between humans. This hypothesis was later confirmed when Walter Reed and his colleagues experimentally confirmed that *Aedes aegypti* mosquitoes were the primary vectors of transmission.

In 1927, YFV became the first mosquito-borne virus to be successfully isolated from the blood of a Ghanaian patient.^130^ A decade later, the live-attenuated 17D vaccine (YF17D) was developed and remains one of the most effective viral vaccines ever produced. However, its efficacy is limited in young children and immunocompromised individuals, leaving these populations at increased risk of infection in endemic areas.^131^ Despite vaccination efforts, yellow fever outbreaks continue to occur in sub-Saharan Africa and tropical South America, often driven by low vaccination coverage, urbanisation, and vector proliferation. To date, no antiviral treatment has been approved for YFV infection.^132^

## Inhibitors of YFV

Recent *in silico* investigations have revealed several promising small molecules that target key enzymes involved in YFV replication, including the RdRp, the NS2B–NS3 protease complex, and the NS1 protein. Among these, Sofosbuvir, a nucleotide analogue initially developed as a prodrug for the Hepatitis C virus, has emerged as a potential inhibitor of YFV replication. Sofosbuvir's active metabolite, triphosphate GS461203, is known to inhibit the RdRp domain of hepatitis C virus NS5B by acting as a chain terminator during RNA synthesis, thereby halting viral genome replication. Building on this mechanism, Mendes *et al.* conducted a comparative study between the RdRp domains of the Hepatitis C virus and YFV to evaluate their structural and functional similarity. Sequence analysis revealed a high level of amino acid conservation, while 3D structural modelling using I-TASSER revealed a moderate similarity with an RMSD of 3.5 Å. Using Schrödinger's PyMOL, the authors aligned the hepatitis C virus RdRp-Sofosbuvir complex (PDB ID: 4WTG) with the predicted YFV RdRp model and found that the critical binding residues were largely conserved, except for a single substitution, Phe224 in the Hepatitis C virus replaced by Trp539 in YFV. Both residues are hydrophobic, maintaining a similar binding pocket environment. These structural and functional parallels, together with prior *in vitro* and clinical evidence supporting Sofosbuvir's antiviral activity, highlight its potential for repurposing as a therapeutic candidate against YFV infection.^133^

In parallel, several studies have focused on the YFV NS3 protease, a key enzyme responsible for cleaving viral polyproteins into functional units essential for replication. Although the NS3 protease typically functions in coordination with the NS2B cofactor, the catalytic activity resides primarily within the NS3 domain, making it an independent and valuable antiviral target. Serafim *et al.* explored this enzyme by investigating a library of 111 aminopyrimidine-based peptidomimetics as potential NS3 protease inhibitors.^134,135^ Using SYBYL-X 2.1.1, they conducted a Hologram quantitative structure–activity relationship (HQSAR) analysis to identify key molecular fragments associated with inhibitory activity, incorporating atomic composition, bond connectivity, chirality, and hydrogen donor–acceptor properties. The best-performing models, characterised by high *q*^2^ values, highlighted structural motifs crucial for activity. To enhance the predictive accuracy, the authors mapped conserved active-site residues across related flaviviral proteases (ZIKV and DENV) and MD simulations to refine structural insights. A pharmacophore model derived from these analyses was then used to screen 7.2 million compounds from multiple chemical libraries. Docking and MD refinement of top hits identified six nonpeptidic ligands with high binding affinity, three of which (compounds 128, 136, and 158) exhibited notable *in vitro* antiviral activity against YFV, demonstrating both the robustness of the virtual screening pipeline and the potential for cross-flavivirus inhibition.^136^

Beyond enzymatic proteases, the YFV NS1 protein is considered a critical target for antiviral intervention due to its dual role in viral replication and immune evasion. Therefore, several *in silico* studies have focused on identifying potential NS1 inhibitors using virtual screening and molecular modelling techniques. In one study, a high-throughput virtual screening (HTVS) approach was conducted using the Schrödinger suite to evaluate chemical libraries from Enamine, Asinex, and NCI for their binding potential against YFV NS1. Initial docking analyses identified three promising candidates, Enamine 89 760, Asinex 3276, and NCI 723, with docking scores of −7.71, −7.70, and −6.69 kcal mol^−1^, respectively. Detailed interaction mapping revealed that Enamine 89 760 formed multiple hydrogen bonds with residues Asp49, Trp97, Ser26, and Ile76, and established a π-cation interaction with Lys51, suggesting strong electrostatic and polar contributions to binding. Moreover, Asinex 3276 interacted with the residues Glu46 and Glu103 *via* salt bridges, while Lys21 and His43 participated in hydrogen bonding and π-cation interactions, highlighting a complementary combination of electrostatic and van der Waals interactions. On the other hand, NCI 723 demonstrated stable hydrogen bonding with Val104 and Glu103, providing further support for its high-affinity engagement with the NS1 binding site. Then, MD simulations confirmed that all three ligands maintained stable binding throughout the trajectories, while frontier molecular orbital (HOMO–LUMO) analyses indicated favourable electronic stability and low chemical reactivity. Importantly, all candidates conformed to Lipinski's rule of five and displayed favourable ADMET properties, reinforcing their potential drug-likeness and suitability for further experimental validation.^137^

**Fig. 17:**
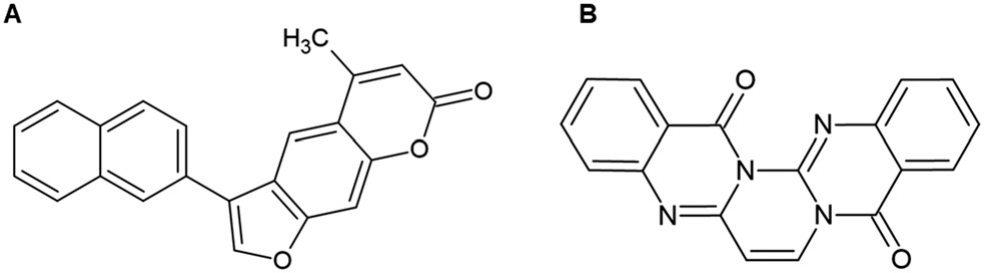
2D structures of the top hits. A) ZINC485867. B) ZINC271839.

Another study focused on identifying novel ligands capable of targeting the NS1 protein across multiple flaviviruses, including YFV, DENV, and ZIKV. Previous research had indicated that the primary binding cavity of NS1 is located within residues 1–28 and 182–216, forming a conserved β-roll structure critical for NS1 dimerisation and function. To ensure structural consistency across the three viruses, the authors selected NS1 monomer conformations that preserved this β-roll motif. The screening of different NS1 conformations was conducted in two sequential steps. First, the viability of potential binding pockets was assessed through visual inspection to identify regions suitable for ligand interaction. Second, these pockets were further filtered using DoGSiteScorer, a computational tool that predicts druggable sites based on pocket shape, volume, and hydrophobicity, allowing selection of high-potential binding cavities. Following pocket selection, a virtual screening campaign was carried out using AutoDock Vina, targeting over 220 000 natural product-derived compounds from the ZINC database. Two ligands, ZINC485867 and ZINC271839, emerged as top candidates (Fig. 17). For the YFV65 NS1 structure, ZINC485867 achieved a docking score of −9.3 kcal mol^−1^, while against YFV162 NS1, it exhibited −10.1 kcal mol^−1^. ZINC271839 scored −9.7 kcal mol^−1^ with the YFV65 NS1 structure. Notably, both compounds exhibited comparable docking affinities across NS1 structures from DENV and ZIKV, which suggests conserved interactions within the β-roll domain. These findings indicate that ZINC485867 and ZINC271839 may function as broad-spectrum, pan-flavivirus inhibitors, capable of targeting NS1 across multiple viral species. By exploiting the conserved β-roll architecture, these ligands highlight the potential for structure-guided discovery of antiviral compounds with cross-flaviviral efficacy.^138^

Overall, these studies highlight the growing impact of integrative *in silico* approaches in identifying potential antiviral candidates against flaviviruses. Despite differences in computational strategies and compound libraries, a common trend emerges in the identification of structurally diverse molecules with promising binding affinities and favourable pharmacokinetic profiles. To provide a consolidated overview of these findings and enable easier comparison across studies, a comprehensive summary is presented in Table 1.

**Table 1:** Summary of computational approaches used to identify flavivirus inhibitors, including targeted viruses and proteins, the computational methods employed, and candidate compounds

Virus	Target protein	Computational methods	Top compounds
ZIKV	NS2B–NS3 protease	Al-based QSAR, virtual screening	MK-591, JNJ-40418677^139^
ZIKV	NS2B–NS3 protease	Monte Carlo simulation-based QSAR,molecular docking	Amentoflavone, fisetin, isorhamnetin, theaflavin-3-gallate^69^
DENV	NS5 MTase	Virtual screening, MD simulations, MM-PBSA	Isoquercitrin^77^
DENV-2	NS5 MTase	ADMET analysis, molecular docking, MDsimulations	CHEMBL376820^78^
DENV	NS3 protease–helicase	Molecular docking, MD simulations, MM-GBSA, ADMET analysis	CID-440015, CID-7424^140^
DENV	NS2B–NS3 protease, NS2 polymerase	Molecular docking, ADMET analysis	Agathisflavone, pectolinarin^80^
DENV	NS2B–NS3 protease	Pharmacophore modelling, virtual screening, QSAR, molecular docking	ZINC36596404, ZINC22973642^83^
DENV-2	NS2B–NS3 protease	QSAR, MLG-GFA model, molecular docking	Compound 21^87^
WNV	NS2B–NS3 protease	Molecular docking	Spiropyrrolidine 5c^108^
WNV, DENV,ZIKV	NS2B–NS3 protease	Molecular docking, MD simulations	Compound 11q^110^
WNV	NS5 MTase, E glycoprotein	ADMET analysis, molecular docking, MD simulations, MM-PBSA	ApigeninFungisterol, sanguinarine^111^
WNV	E glycoprotein	Molecular docking, MD simulations, MM-GBSA	Cianidanol, L-rhamnose^112^
WNV, DENV	NS5 MTase	AutoQSAR, molecular docking	CID439610^113^
JEV	NS2B–NS3 protease	Molecular docking, QSAR	CW-33^120^
JEV	NS3 protease	Molecular docking, MD simulations	Andrographolide^122^
JEV	NS5RdRp, NS5MTase	Molecular docking, MD simulations	Withaferin A^123^
JEV	NS3, RdRp, NS5, E glycoprotein, C protein	Virtual screening, molecular docking, ADMET analysis, MD simulations	3-isobutyl-2,3,6,7,8,8a-hexahydropyrrolo [1,2] pyrazine-1,4-dione(Comp-A), Pyrrolo[1,2-*a*]pyrazine-1,4-dione,hexahydro-3-(phenylmethyl)- (Comp-B), 2,4-di-*tert*-butylphenol(Comp-C)^127^
ZIKV, DENV-2 & 3, YFV	NS3 protease	HQSAR, molecular docking, MD simulations	Compounds 136 & 154, raloxifene^136^
ZIKV, DENV, YFV	NS1	Md simulations, virtual screening, molecular docking, ADMET analysis	ZINC485867, ZINC271839^138^

## Limitations and challenges of computational approaches

Despite the rapid expansion of *in silico* methodologies and significant advances in computational drug discovery within antiviral research, these approaches remain subject to inherent methodological limitations that can compromise the reliability and translational success of predicted inhibitors. One of the primary challenges of the molecular docking approach is the simplification of protein–ligand interactions, which often fails to fully capture the dynamic conformational flexibility of flavivirus proteins and the complex nature of binding processes in biological systems. Most docking algorithms employ rigid or semi-flexible receptor assumptions and rely on scoring functions that approximate binding free energy. These scoring functions often fail to accurately capture key physicochemical contributions such as solvent effects, entropic penalties, induced-fit phenomena, and long-range electrostatics.^141^ As a result, docking scores do not consistently correlate with experimentally determined binding affinities, leading to a high rate of false positives in virtual screening campaigns. Consequently, docking outputs should be interpreted as qualitative ranking tools rather than quantitative predictors of biological activity. To partially address these limitations, many studies integrate MD simulations and MM-PB(GB)SA-based binding free energy calculations as downstream refinement steps to account for protein flexibility and time-dependent ligand–protein interactions. These approaches capture dynamic conformational states that can reveal hidden or transient binding sites inaccessible in static structures or mature protein particles. Moreover, protein dynamics across a broad range of timescales (picoseconds to milliseconds) can be investigated using advanced MD techniques.^142^ Cosolvent simulations (*e.g.*, SWISH, MixMD, and CrypticScout) further facilitate the identification of binding hotspots within cryptic pockets, while enhanced sampling methods such as replica exchange and metadynamics, when combined with ensemble docking and free-energy calculations, significantly improve predictive accuracy.^143–145^ Although these approaches provide improved insight into pocket identification, ligand stability, and dynamic behaviour compared to docking alone, their predictive accuracy is still constrained by force field approximations and sensitivity to system preparation parameters. Furthermore, MD simulations are often performed on single protein–ligand trajectories under idealised conditions, which may not fully represent the complexity of physiological environments. Therefore, binding free energy estimates derived from these methods should be considered comparative rather than absolute thermodynamic values, particularly when used across structurally diverse ligand sets.

Moreover, the reliability of both MD-based and docking-based predictions is fundamentally dependent on the structural quality of the target protein used as input. In many antiviral studies, experimentally resolved crystal structures are unavailable, leading to the widespread use of homology models for structure-based screening. While homology modelling is valuable for enabling computational investigations in the absence of experimental structures, its accuracy is strongly influenced by template selection, sequence identity, and the reliability of loop and active-site region reconstruction. As a result, structural inaccuracies in key binding-site geometries can propagate through downstream docking and MD workflows, ultimately affecting predicted binding poses, interaction profiles, and affinity rankings. This introduces an additional and often underappreciated layer of uncertainty in computational drug discovery pipelines.^146^ Beyond structure-dependent approaches, data-driven methodologies such as QSAR models and molecular fingerprint-based similarity methods introduce a different but equally important set of limitations. Although these approaches provide powerful predictive frameworks for prioritising compounds and exploring chemical space, their performance is highly dependent on the diversity, size, and quality of the training dataset used for model construction. When applied to compounds that fall outside the chemical space represented in the training data, model generalisability can decrease significantly, leading to reduced predictive reliability.^147^ Similarly, fingerprint-based similarity metrics may fail to capture subtle yet biologically relevant structural and physicochemical differences, particularly in cases involving scaffold hopping or novel chemical scaffolds.

Taken together, these limitations reflect a broader challenge in computational antiviral research, namely the lack of standardisation across methodological workflows. Across the literature, differences in docking software, scoring functions, protein preparation protocols, ligand protonation states, grid parameters, and MD simulation settings make direct comparison of binding affinities across studies challenging. This methodological variability reduces reproducibility and limits the ability to quantitatively rank inhibitors reported from different computational pipelines, even when targeting the same viral protein. This lack of standardisation is further compounded by the limited extent of experimental validation. Although many studies report promising virtual hits, only a small proportion proceed to *in vitro* or *in vivo* testing. This creates a bias in the literature toward computationally “successful” compounds that may not necessarily translate into biologically active or clinically relevant antivirals. Therefore, computational predictions should be considered hypothesis-generating tools that require rigorous experimental confirmation before therapeutic relevance can be established.

## Future perspectives

A key future direction lies in improving the physiological relevance of computational models. Incorporating protein flexibility through ensemble docking, longer and replicate MD simulations, and advanced free energy methods (*e.g.*, alchemical calculations) could provide more robust and reliable estimates of ligand-target interactions. In parallel, greater use of experimentally resolved protein conformations, particularly high-resolution cryo-EM structures, and continuously updated homology modelling pipelines will enhance the structural accuracy of docking inputs and reduce uncertainty propagated through downstream analyses. Furthermore, machine-learning-based structure prediction tools such as AlphaFold are rapidly advancing and already demonstrate the ability to capture certain conformational changes. As viral mutation rates continue to accelerate during epidemics, increasingly accurate AI-driven predictions of structural variation and its impact on binding-site accessibility will be essential for the timely development of effective antiviral therapies.

From an expert perspective, future antiviral research should increasingly prioritise multi-target drug design and modulation of virus-host interfaces. Targeting multiple viral enzymes, such as the NS2B/NS3 protease and NS5 polymerase, or simultaneously interfering with essential host factors required for viral replication, may yield more robust therapeutic strategies and reduce the likelihood of resistance development. In addition, the integration of AI-driven *de novo* drug design with structure-based modelling offers a powerful opportunity to identify novel chemical scaffolds with improved antiviral potency and pharmacokinetic properties. In this context, deep learning models trained on large-scale bioactivity datasets, combined with molecular fingerprinting and graph-based neural networks, may significantly enhance predictive performance beyond traditional QSAR approaches. However, the success of these methods remains strongly dependent on rigorous dataset curation and robust external validation to ensure generalisability across diverse chemical spaces and viral targets.

Another critical consideration for future progress is the standardisation of computational workflows. The establishment of community-wide guidelines for docking protocols, MD simulation parameters, and binding free energy calculations would substantially improve reproducibility and enable more meaningful cross-study comparisons. Likewise, the development of open-access benchmarking datasets for flavivirus targets would provide a valuable resource for objectively evaluating computational methods, scoring functions, and predictive accuracy. Finally, closer integration between computational predictions and experimental validation remains essential for translational success. Future antiviral discovery pipelines should adopt iterative feedback frameworks in which *in silico* predictions are continuously refined using biochemical, cellular, and *in vivo* experimental data. Such an integrated approach will help bridge the gap between computationally identified hits and clinically relevant antiviral candidates, ultimately accelerating the development of effective therapeutics against flavivirus diseases.

## Conclusion

Flaviviruses remain a significant global health challenge due to their widespread prevalence, capacity to cause severe disease, and frequent emergence of drug-resistant strains. Despite the availability of vaccines for some flaviviruses, effective antiviral therapies are still limited, emphasising the urgent need for novel therapeutic strategies. The structural and functional diversity of flavivirus proteins, including both structural and NS components, provides multiple promising targets for drug development. Computational methodologies have proven invaluable in identifying and optimising inhibitors with improved selectivity and potency. The studies reviewed herein demonstrate that *in silico* approaches can successfully guide hit discovery, predict binding affinities, and refine pharmacokinetic properties, thereby significantly reducing experimental time and cost. Integrating computational techniques with experimental validation represents a robust framework for advancing antiviral drug discovery. Moving forward, the continued application and refinement of these strategies, alongside exploration of novel viral and host targets, will be critical in developing safe and effective therapeutics to mitigate the global burden of flavivirus infections.

## Conflicts of interest

There are no conflicts to declare.

## Data Availability

All primary data supporting the findings of this study are presented within the manuscript.
